# Diversity and antimicrobial potential in sea anemone and holothurian microbiomes

**DOI:** 10.1371/journal.pone.0196178

**Published:** 2018-05-09

**Authors:** Elizabeth León-Palmero, Vanessa Joglar, Pedro A. Álvarez, Antonio Martín-Platero, Inmaculada Llamas, Isabel Reche

**Affiliations:** 1 Departamento de Ecología and Instituto del Agua, Universidad de Granada, Granada, Spain; 2 iMare Natural S.L., Motril, Granada, Spain; 3 Departamento de Microbiología, Universidad de Granada, Granada, Spain; 4 Instituto de Biotecnología, Centro de Investigaciones Biomédicas (CIBM), Universidad de Granada, Granada, Spain; Pusan National University, REPUBLIC OF KOREA

## Abstract

Marine invertebrates, as holobionts, contain symbiotic bacteria that coevolve and develop antimicrobial substances. These symbiotic bacteria are an underexplored source of new bioactive molecules to face the emerging antibiotic resistance in pathogens. Here, we explored the antimicrobial activity of bacteria retrieved from the microbiota of two sea anemones (*Anemonia sulcata*, *Actinia equina*) and two holothurians (*Holothuria tubulosa*, *Holothuria forskali*). We tested the antimicrobial activity of the isolated bacteria against pathogens with interest for human health, agriculture and aquaculture. We isolated 27 strains with antibacterial activity and 12 of these isolates also showed antifungal activity. We taxonomically identified these strains being *Bacillus* and *Vibrio* species the most representative producers of antimicrobial substances. Microbiome species composition of the two sea anemones was similar between them but differed substantially of seawater bacteria. In contrast, microbiome species composition of the two holothurian species was different between them and in comparison with the bacteria in holothurian feces and seawater. In all the holobiont microbiomes *Bacteroidetes* was the predominant phylum. For each microbiome, we determined diversity and the rank-abundance dominance using five fitted models (null, pre-emption, log-Normal, Zipf and Zipf-Mandelbrot). The models with less evenness (i.e. Zipf and Zipf-Mandelblot) showed the best fits in all the microbiomes. Finally, we tracked (using the V4 hypervariable region of 16S rRNA gene) the relative abundance of these 27 isolates with antibacterial activity in the total pool of sequences obtained for the microbiome of each holobiont. Coincidences, although with extremely low frequencies, were detected only in the microbiome of *H*. *forskali*. This fact suggests that these isolated bacteria belong to the long tail of rare symbiotic bacteria. Therefore, more and more sophisticated culture techniques are necessary to explore this apparently vast pool of rare symbiontic bacteria and to determine their biotechnological potentiality.

## Introduction

Worldwide, microbial pathogens, including bacteria and fungi, are responsible for multiple diseases. These infections affect not only humans but also crops, livestock, and aquaculture generating the need of new antimicrobial agents, in part, due to the increase in the antibiotic resistance of some pathogens [1]. For instance, the fungus *Botrytis cinerea* provokes thousands of tons of losses in the wine industry. The use of microbial pesticides (microbes or their metabolites) has been suggested as a sustainable alternative to face this infection [2]. Similarly, in aquaculture, the fastest-growing food-producing sector [3], the disease outbreaks in the tanks or cages is one of the most recurrent problems [4]. For example, *Vibrio* species are responsible of vibriosis, a deadly hemorrhagic septicemia disease with high morbidity and mortality rates in fish, bivalves, crustaceans and corals. In aquaculture facilities, to control potential bacterial diseases, antibiotics have been routinely used as daily prophylactic doses [5]. This uncontrolled and frequent use of antibiotics has resulted in the development of resistance in several pathogens. For this reason, antibiotics are no longer effective in some cases [6]. On the other hand, this worldwide increase in Vibrio-associated diseases could be also related to global change issues [7]. In any case, irrespectively of its origin, new antimicrobial products are necessary to control pathogenic bacteria in this particular food sector and to face the antibiotic resistance problem in general [8,9].

Marine biodiversity is an underexplored source of new bioactive products [10–12]. Therefore, it is expected that the discovery of new antimicrobial compounds from these marine invertebrates will provide new and better therapeutics for human illnesses, along with other bioactive products for a sustainable production of food [13–15]. Since the first bioactive molecules were discovered in the sponge *Cryptotethya crypta* [16,17], the microbiota of marine invertebrates have been deeply studied and every year new substances are isolated [18]. All animals are holobionts containing symbiotic microorganisms that evolve jointly [19–21] and create complex consortia [22]. These microorganisms colonize the animal skin, the mucosal surfaces and the gut. Different functions, from nutrient provision to infection prevention, have been attributed to this symbiotic microbiota [19]. Holobionts have co-evolved with their microbiota developing potent protective mechanisms with antimicrobial activity, likely associated to secondary metabolites [23–25]. This antimicrobial activity produced by the symbiotic bacteria is usually associated to uncultured species. This pool of bacteria, known as the microbial dark matter, is now being intensively investigated with biotechnology purposes [1,9,24,26]. Marine invertebrates, the benthonic animals in particular, due to their soft body and sessile lifestyle, rely on chemical defenses to protect themselves against competitors, predators and infectious microorganisms. Therefore, they are excellent candidates for the search of symbiotic bacteria with antimicrobial potential. Cnidarians, as soft corals, gorgonians and sea anemones, have a high diversity of symbiotic bacteria [27,28], especially as the coral surface is more nutrient-rich than seawater or sediments [29,30]. Indeed, some authors have detected coral-associated bacteria as sources of antimicrobial products [27,31–33]. Other works have also reported antimicrobial, particularly antifungal, activity in echinoderm microbiota [25, 34–38]. In the case of holothurians, different studies have also shown that they have an unique microbiota [39,40], but their role as antimicrobial producers has been seldom studied [23,41].

The relative importance of bacteria with antimicrobial potential within the whole pool of symbiotic bacteria in marine invertebrate is practically unknown. The current advance in sequencing technology is making possible to explore microbiome thoroughly, beyond the biased information obtained by pure culture techniques [42,43]. Recurrently, in natural ecosystems, including holobiont microbiomes, most bacteria belong to a few operational taxonomic units (OTUs); which contain a high number of representatives named “core” species. In contrast, the vast majority of bacterial OTUs in natural ecosystems includes only a few representatives and these OTUs are named “rare” species [43–46]. Therefore, the knowledge on the relative importance of the bacteria with antimicrobial potential in the whole pool of symbiotic bacteria in marine invertebrates can be essential to improve and optimize their exploration for biotechnological and medical purposes.

In this study, we isolated and identified bacterial strains with antimicrobial activity against pathogens of humans (*Staphylococcus aureus*, *Pseudomonas aeruginosa*), plants (including bacteria: *Erwinia amylovora*, *Agrobacterium tumefaciens* and fungus: *Botrytis cinerea*, *Verticillium dalhliae*, *Phytium ultimum*, *Phytophthora cinnamomi*, *Thanatherophorus cucumeris*, *Magnaporthe oryzae* and *Sclerotinia sclerotiorum*) and fishes and shellfish from aquaculture (*Vibrio brasiliensis*, *V*. *anguillarum*, *V*. *mediterranei*, *V*. *coralliilyticus*). The source of these isolates was the microbiota of two sea anemones (*Actinia equina* and *Anemonia sulcata*) and two sea cucumbers (*Holothuria tubulosa* and *H*. *forskali*). In addition, we determined the microbiota diversity in these holobiont microbiomes and the relative importance of these bacterial strains with antimicrobial potential in the whole microbiome.

## Material & methods

### Collection of samples

We collected the samples at iMARE Natural S.L. facilities (Motril, Granada, Spain). All living animals and feces were taken from the same aquaculture tank. The sea anemone species were *Anemonia sulcata* and *Actinia equina* (class *Anthozoa*, order *Actiniaria*, family *Actiniidae*). We took from 4 specimens of *Anemonia sulcata* the tentacles (sample M1) and the guts (sample M2). We also dissected 7 specimens of *Actinia equina*, taking the tentacles (sample M7) and the guts (sample M8). The selected holothurian species were *Holothuria tubulosa* and *Holothuria forskali* (class *Holothurioidea*, order *Aspidochirota*, family *Holothuriidae*). We took the intestines (sample M3) and the coelomic fluids (sample M4) from 2 specimens of *Holothuria tubulosa*. We also took the intestines (sample M5) and the coelomic fluids (sample M6) from 2 specimens of *Holothuria forskali*. In addition, we collected samples of holothurian feces (Sample M9) and seawater (sample M10) of the aquaculture tank. Every sample was homogenized and divided in two fractions. The first fraction was used to obtain the pure cultures of the symbiotic bacteria of these marine holobionts and to test their antimicrobial potential. The second fraction was used to characterize the whole microbiome by massive sequencing of the V4 hypervariable region of 16S rRNA gene and, subsequently, to determine the relative abundance of the cultured bacteria with antimicrobial potential in the whole microbiome. Seawater sample (M10) was used only to determine the bacterial assemblage in the aquaculture tank as a control.

### Pure culture of bacteria from the holobiont microbiotas

We performed serial dilutions up to 10^−5^ using one fraction of the homogenized samples using a sterile 1% (w/v) NaCl solution. A volume of 100 μL of each dilution (i.e. 10^−1^, 10^−2^, 10^−3^, 10^−4^ and 10^−5^) was surface-plated on marine agar medium (MA, Difco). The plates were incubated at 26°C for 7 days. A collection of 827 isolates, randomly chosen, were re-isolated by streaking on a fresh medium and incubated at 26°C for 48–72 hours. Cultures of each isolate in marine broth (MB, Difco) (200 μL) were kept in 96-well microtiter plates. To each well 25μL of glycerol 80% (v/v) were added to store them at -80°C.

### Indicator strains

To test the antibacterial activity we used the next indicator strains: *Staphylococcus aureus* ATCC 25923, *Pseudomonas aeruginosa* ATCC 15692, *Erwinia amylovora* ATCC 49946, *Agrobacterium tumefaciens* ATCC 33970, *Vibrio brasiliensis* LMG 20546, *Vibrio anguillarum* ATCC 68554, *Vibrio mediterranei* ATCC 43341, and *Vibrio coralliilyticus* ATCC BAA450. *S*. *aureus*, *E*. *amylovora*, and *P*. *aeruginosa* were cultured at 30° C in Luria Bertani medium (LB, Panreac). *A*. *tumefaciens* was cultured at 30° C in LB supplemented with CaCl_2_·2H_2_O (2.5 mM) and MgSO_4_·7H_2_O (2.5 mM). *Vibrio* species were cultured at 26°C in MB.

To test the antifungal activity we used the next indicator strains: *Botrytis cinerea* CECT 2850, *Alternaria alternata* CECT 2662, *Verticillium dalhliae* CECT 2694, *Fusarium oxysporum* CECT 2154, *Phytium ultimum* CECT 2364, *Phytophthora cinnamomi* CECT 20186, *Thanatherophorus cucumeris* CECT 2813, *Magnaporthe oryzae* CECT 20276 and *Sclerotinia sclerotiorum* CECT 2769. Fungi were cultured at 25° C in Potato Dextrose Agar (PDA, Prolabo) medium.

### Antibacterial and antifungal activity of isolated strains

We carried out a pre-screening test using agar plates (135 mm diameter) of MA and TSA 1% (w/v) NaCl media. The plates were overlaid with 40 mL of 0.7% (w/v) agar containing either overnight culture of *Staphylococcus aureus* ATCC 25923 or *Vibrio brasiliensis* LMG 20546 at a density of 10^6^ colony-forming units (CFU) mL^-1^. After 30 minutes, sterilized 96-well microtiter replicator was submerged in each master microtiter plate containing the isolates in MB and spotted onto the agar surface. Screening plates were incubated at 30°C for 48–72 hours and the strains with inhibition halo were identified to continue the screening test (S1 Fig.)

We carried out a second screening test using a well diffusion agar-plate technique [47] placing stainless steel cylinders over a LB or MA plates. Then, the plates were overlaid with 10 mL of 0.7% (w/v) agar containing an overnight culture of an indicator bacterium at a density from 10^6^ to 10^7^ CFU mL^-1^. During this test we used the culture supernatant, therefore we detected only the bacteria that secreted antibacterial substances to the environment during their growth, before being exposed to the pathogenic microorganism. In this second step, the selection criterion was stricter. The indicator strains used were *Staphylococcus aureus* ATCC 25923, *Pseudomonas aeruginosa* ATCC 15692, *Erwinia amylovora* ATCC 49946, *Agrobacterium tumefaciens* ATCC 33970, *Vibrio brasiliensis* LMG 20546, *V*. *anguillarum* ATCC 68554, *V*. *mediterranei* ATCC 43341, and *V*. *coralliilyticus* ATCC BAA450. The cylinders were removed when the overlayer was solidified, and 100 μL of each sample supernatant was pipetted into each well. Supernatants were obtained after centrifugation at 10,000 rpm for 3 minutes of marine bacterium cultures in MB or LB broth incubated at 26°C and agitated at 100 rpm for 48–72 hours. Plates were incubated at 30°C for 48–72 hours and the inhibition halos around wells were identified (S2 Fig) and measured (S1 Table).

The antifungal activity of the isolates was tested against phytopathogen fungi in Potato dextrose broth medium (PDB) using the services of the company Xtreme Biotech (http://www.xtrembiotech.com), a spin-off company of the University of Granada. The fungus cultures were homogenized with a sterilized commercial blender. Streptomycin (10 mg L^-1^) and penicillin G (2.5 mg L^-1^) were added to the homogenized culture. Isolates were cultured at 26°C in MB for 48–72 hours. Then, cultures were centrifuged at 10,000 rpm for 3 minutes. The antifungal screening test was performed using 48-well microtiter plates (1.7 mL per well). The fungal culture (1.2 mL with 10^6^ CFU mL^-1^) and the supernatant obtained from cultured bacteria (0.400 mL) were deposited in each well. The negative control consisted of fungal culture with cycloheximide (50 mg mL^-1^). The positive control was fungal culture without cycloheximide. Plates were incubated at 28°C for 30 days. The results were examined every week during 30 days by measuring the turbidity of each culture (S3 Fig).

### Extraction, amplification, and sequencing of 16S rRNA gene of the bacteria with antimicrobial activity

We extracted the DNA following the procedure proposed by Martin-Platero et al. (2007) [48] modified from Miller et al. 1988 [49]. Then, the 16S rRNA gene was amplified by the PCR using standard protocols [50]. The forward primer was 16F27 (5’-AGAGTTTGATCATGGCTCAG-3’) and the reverse primer was 16R1488 (5’-CGGTTACCTTGTTAGGACTTCACC-3’)[51]. PCR amplifications were made using 50 μL of reaction mixtures containing 20–100 ng of template DNA, 20 pmol of each primer (Sigma), 0.2 mmol L^-1^ dNTP mix (Bioline), 2 mmol L^-1^ MgCl_2_, 5 μL of PCR buffer (10×) (Bioline), and 1.25 U of BIOTAQ DNA polymerase (Bioline). PCR products were purified using the ISOLATE II PCR and Gel Kit (Bioline) according to the manufactures’ recommendation. DNA concentration was measured and adjusted according the Genetic Analyzer Sequencer requirements. The direct sequencing of PCR-amplified DNAs was carried out using the Applied Biosystems 3730XL Genetic Analyzer Sequencer (HITACHI) at Macrogen Europe facilities in Amsterdam (Netherlands). Three universal primers were used for each 16S rRNA gene sequence: forward 16F27, reverse 16R1488 and an intermediate primer, U515 (GTGCCAGCMGCCGCGGTAA) that is specific for the V4 hypervariable region of this gene. The reads were assembled to obtain the bacterial 16S rRNA gene consensus sequences of varying lengths between 958 and1571 bp using Geneious version (9.0.5) (http://www.geneious.com) [52]. To taxonomically identify the isolates, the sequences were compared to 16S rRNA gene sequences available in the GenBank and EMBL databases obtained from the National Center for Biothecnology Information (NCBI) database by the BLAST (http://blast.ncbi.nlm.nih.gov/Blast.cgi) algorithm at EzTaxon server (http://www.ezbiocloud.net/eztaxon) [53]. The sequences obtained were deposited in GenBank/EMBL/DDBJ with accession number from KX369290 to KX369316.

### Extraction, amplification and massive sequencing of the V4 region of the 16S rRNA gene in the holobiont microbiomes

We used the second fraction of the homogenized samples for massive sequencing of only the V4 region of 16S rRNA gene in the microbiomes. DNA extraction was carried out following the procedure described in the commercial kit FavorPrep™. The medium length of DNA fragments was determined by electrophoresis in agarose gel (0.7% w/v). DNA concentration was measured and normalized. The V4 hypervariable region of 16S rRNA gene was amplified by PCR using universal primers U515 and E786F (GTCTCGTGGGCTCGGAGATGTGTATAAGAGACAGGGACTACHVGGGTWTCTAAT) with overhang partial Illumina adapters. The products were purified with the *GenElute™PCR Clean-Up* (Sigma) commercial kit, according to the manufactures’ recommendation. Then, a couple of two unique Illumina compatible barcodes was added to each sample using *Nextera XT DNA Library Preparation* Kit (Illumina); which allows the preparation of sequencing-ready libraries. They were mixed to create the sequencing library. A cleaning step, using a commercial kit, and quantification was repeated. The sequencing of PCR-amplified DNAs with the barcodes was performed using the MiSeq Illumina sequencing platform at the Centro de Instrumentación Científica of the University of Granada. The sequencing run consisted of pair-end reads of 250 bp. For data analysis, the forward and reverse fastq files were concatenated, filtered by read quality (Phred Score) and the barcodes and primers sequences were eliminated using QIIME [54] and MOTHUR [55]. The final length of V4 sequences was ∼252 bp. For quality filtering, QIIME default parameters were used, with a minimun Phred quality score <20 and ambiguous nucleotides were removed. Sequence alignment was made using Greengenes database [56] and PyNast alignment algorithm [57]. UCLUST software [58] was used to assign similar sequences to operational taxonomic units (OTUs) by clustering sequences based on a 97% similarity threshold. The taxonomic assignment was achieved in different levels of resolution, from phyla to genera or species. UCLUST was used to chimera removal. The unassigned OTUs obtained were assigned using RDP’s Classifier [59] release 11.4 (May 26, 2015). The FASTQ files generated after the metagenome sequencing were deposited in Sequence Read Archive database (SRA-NCBI; https://www.ncbi.nlm.nih.gov/sra), with the BioProject ID PRJNA420053 and BioSample accession numbers from SAMN08104840 to SAMN08104849.

### Data analysis of the holobiont microbiomes

To obtain microbiome diversity of the holobionts we merged in one data set the sequences of tentacles and gut of each corresponding sea anemones (i.e. M1+M2 sequences and M7 +M8 sequences). Similarly, the sequences the intestines and coelomic fluid of each holothurian species were pooled in just one data set (i.e. M3+M4 sequences and M5+M6 sequences). Diversity analysis were performed in R [60] using the Vegan package [61]. To determine the shape of OTUs abundance distribution we tested five models: brokenstick (null), pre-emption, log-Normal, Zipf and Zipf-Mandelbrot. These Rank-Abundance Dominance (RAD) curves display logarithmic species abundances against rank order [62]. The model with the best fit (according to the Akaine’s Information Criterion (AIC) was selected. The smaller the AIC, the better the fit is [63].

To determine the relative importance of the cultivable bacteria with antimicrobial activity, we tracked their V4 hypervariable sequences in the total pool of V4 sequences in each corresponding holobiont microbiomes. We used different similarity identity thresholds using USEARCH tool from Galaxy Platform [64]. This tool is based on USEARCH and UCLUST algorithms [58]. These thresholds varied from 100% to 98.00%, i.e. from 0 to 5 different nucleotides in the sequences aligned (252 bp length). The coincidence frequency is the number of coincident sequences at a given level of similarity (for instance, 100% similarity) divided by the total pool of sequences obtained with the Illumina platform for that particular sample.

## Results

### Isolation and selection of symbiotic bacteria with antimicrobial activity

In a first step, 827 isolates from the microbiome of the sea anemones *Anemonia sulcata* and *Actinia equina* (tentacles and gut) and the sea cucumbers *Holothuria tubulosa* and *H*. *forskali* (intestines, coelomic fluid and feces) were randomly selected and kept in 96-well microtiter plates. We performed a pre-screening test of the antibacterial activity only against the pathogens *Staphylococcus aureus* ATCC 25923 and *Vibrio brasiliensis* LMG 20546. Then, we selected 193 strains that produced inhibition halo against one or both pathogens (S1 Fig).

In a second screening, we performed a well diffusion agar-plate assay. This assay consisted in placing stainless steel cylinders (Oxford towers) of the 193 selected strains in the presence of the pathogens: *Staphylococcus aureus* ATCC 25923, *Pseudomonas aeruginosa* ATCC 15692, *Erwinia amylovora* ATCC 49946, *Agrobacterium tumefaciens* ATCC 33970, *Vibrio brasiliensis* LMG 20546, *V*. *anguillarum* ATCC 68554, *V*. *mediterranei* ATCC 43341, and *V*. *coralliilyticus* ATCC BAA450. These assays were carried out in triplicate. Using this technique, we selected 27 out of the 193 strains with antibacterial activities against different pathogenic bacteria (S2 Fig). We obtained one isolate (M9-44) that showed inhibitory activity against five out of 8 pathogenic bacteria tested, three isolates (M2-16-2; M2-61 and M9-53-1) that showed inhibitory activity against four of the pathogenic bacteria tested and five isolates (M6-45; M8-1; M8-6; M9-11 and M9-61) against three of the pathogenic bacteria tested. The isolates showed predominant inhibitory activities against *E*. *amylovora* ATCC 49946 (20 isolates), *S*. *aureus* ATCC 25923 (19 isolates) and *A*. *tumefaciens* ATCC 33970 (7 isolates) (Table 1). These 27 strains with antibacterial activity were obtained from coelomic fluid of *Holothuria forskali* (seven), from coelomic fluid of *H*. *tubulosa* (one) and from holothurian feces (six). Sea anemones microbiota also showed a relevant antimicrobial potential, with two strains obtained from the tentacles and five from the gut of *Actinia equina*. Two strains were obtained from the tentacles and three strains from the gut of *Anemonia sulcata* (Table 1).

**Table 1:** Antibacterial activity of the 27 isolated bacteria from *Anemonia sulcata* (samples M1, M2), *Holothuria tubulosa* (M3, M4), *Holothuria forskali* (M6), *Actinia equina* (M7, M8) and holothurian feces (M9) against eight pathogenic bacteria.

Sample	Isolate ID	*Staphylococcus aureus*	*Erwinia amylovora*	*Pseudomonas aeruginosa PAO1*	*Agrobacterium tumefaciens*	*Vibrio brasiliensis*	*Vibrio anguillarum*	*Vibrio mediterranei*	*Vibrio coralliilyticus*	Total
M1: *Anemonia sulcata* (tentacles)	M1-5-2	+	-	-	-	-	+	-	-	2
	M1-33	+	+	-	-	-	-	-	-	2
M2: *Anemonia sulcata* (gut)	M2-12	+	+	-	-	-	-	-	-	2
	M2-16-2	+	+	-	-	-	+	+	-	4
	M2-61	+	+	-	-	-	+	+	-	4
M3: *Holothuria tubulosa* (intestines)	M3-59	+	-	-	+	-	-	-	-	2
M4: *Holothuria tubulosa* (coelomic fluid)	M4-71	+	-	-	-	-	+	-	-	2
M6: *Holothuria forskali* (coelomic fluid)	M6-1	-	+	-	-	-	-	-	-	1
	M6-12-2	+	-	+	-	-	-	-	-	2
	M6-25	+	-	-	-	-	-	-	-	2
	M6-26-1	-	+	-	-	-	-	-	-	1
	M6-26-2	-	+	-	-	-	-	-	-	1
	M6-33	-	+	-	+	-	-	-	-	2
	M6-45	+	+	-	+	-	-	-	-	3
M7: *Actinia equina (*tentacles*)*	M7-11-1	+	+	-	-	-	-	-	-	2
	M7-11-2	+	+	-	-	-	-	-	-	2
M8: *Actinia equina (*gut*)*	M8-1	+	+	-	+	-	-	-	-	3
	M8-2	+	-	-	+	-	-	-	-	2
	M8-6	+	+	-	+	-	-	-	-	3
	M8-15	+	-	-	-	-	-	-	-	1
	M8-24-1	+	+	-	-	-	-	-	-	2
M9: *H*. *tubulosa* and *H*. *forskali* (feces)	M9-11	+	+	-	+	-	-	-	-	3
	M9-27-1	-	+	-	-	-	-	-	-	1
	M9-44	-	+	-	-	+	+	+	+	5
	M9-53-1	+	+	-	-	-	+	+	-	4
	M9-53-2	-	+	-	-	-	-	+	-	2
	M9-61	-	+	-	-	-	+	+	-	3
	Total	19	20	2	7	1	7	6	1	

– no antibacterial activity; + inhibitory activity observed

To select the bacteria with antifungal activity, we only selected randomly 86 isolates out of the 193 bacteria with antibacterial activity for economical reasons. We tested these 86 isolates against the phytopathogen fungi: *Botrytis cinerea* CECT 2850, *Alternaria alternata* CECT 2662, *Verticillium dalhliae* CECT 2694, *Fusarium oxysporum* CECT 2154, *Phytium ultimum* CECT 2364, *Phytophthora cinnamomi* CECT 20186, *Thanatherophorus cucumeris* CECT 2813, *Magnaporthe oryzae* CECT 20276, and *Sclerotinia sclerotiorum* CECT 2769 These assays were carried out in duplicate and we observed that 66 out of 86 strains showed antifungal activity against at least one pathogen (S3 Fig). We selected the 12 strains that showed the highest antifungal activities (Table 2.). We found one isolated bacterium (M9-44) with inhibitory activity against five out of seven fungal pathogens tested, three isolated bacteria (M2-12, M3-59 and M8-6) against four fungal pathogens and four isolated bacteria (M1-33, M2-16-2, M2-61, and M8-1) against three fungal pathogens. Like in antibacterial screening test, M9-44 was the strain with more antifungal activity. We obtained 9 isolated bacteria that showed inhibitory activity against *Magnaporthe oryzae* and 8 isolated bacteria against *Thanatephorus cucumeris* and *Sclerotinia sclerotiorum* (Table 2).

**Table 2:** Antifungal activity of the 12 selected bacteria from *Anemonia sulcata* (samples M1, M2), *Holothuria tubulosa* (M3, M4), *Actinia equina* (M8) and holothurian feces (M9) against the nine indicator fungi.

Sample	Isolate ID	*Botrytis cinerea*	*Alternaria alternata*	*Verticillium dahliae*	*Fusarium oxysporum*	*Pythium ultimum*	*Phytophthora cinnamomi*	*Thanatephorus cucumeris*	*Magnaporthe oryzae*	*Sclerotinia sclerotiorum*	Total
M1: *Anemonia sulcata* (tentacles)	M1-33	-	-	-	-	-	-	+	+	+	3
M2: *Anemonia sulcata* (gut)	M2-12	-	-	-	-	+	-	+	+	+	4
	M2-16-2	-	-	+	-	-	-	-	+	+	3
	M2-61	-	-	-	-	-	-	+	+	+	3
M3:*Holothuria tubulosa* (intestines)	M3-59	-	-	-	-	-	+	+	+	+	4
M4:*Holothuria tubulosa* (coelomic fluid)	M4-71	-	-	-	-	-	+	*	*	*	1
M8: *Actinia equina (gut)*	M8-1	-	-	-	-	-	-	+	+	+	3
	M8-2	-	-	-	-	-	+	+	-	-	2
	M8-6	-	-	+	-	-	-	+	+	+	4
M9: *H. tubulosa* and *H. forskali* (feces)	M9-27-1	-	-	-	-	-	-	-	+	+	2
	M9-44	+	-	-	-	+	+	+	+	-	5
	M9-61	-	-	-	-	-	+	*	*	*	1
	Total	1	0	2	0	2	5	8	9	8	

– no antifungal activity; + inhibitory activity observed

* no evaluated

### Taxonomical identification of the isolated bacteria with antimicrobial activity

The taxonomical identification of the selected 27 isolated bacteria with antimicrobial activity was based on the 16S rRNA gene sequence (Table 3 and S2 Table). *Bacillus* (phylum *Firmicutes*) was the predominant genus with 18 strains and, particularly, the *Bacillus subtilis* group with 11 strains. *Bacillus pumilus* group (4 strains), *Bacillus marisflavi* TF-11^T^, *Bacillus aerophilus* group and *Bacillus anthracis* group were also identified. The phylum *Proteobacteria*, specifically the Class *Gammaproteobacteria* was represented with 11 strains of the taxonomical group *Vibrio alginolyticus* (belonging to *V*. *alginolyticus* group 5 strains), *Pseudoalteromonas tetraodonis* (5 strains), *Stenotrophomonas maltophilia* group and *Psychrobacter faecalis* group. The same bacterial groups have been found in samples from different holobionts.

**Table 3:** Phylogenetic affiliations of the 27 isolated bacteria with antimicrobial activity using Eztaxon database [53].

Sample	Isolate ID	Accession number	Closest identified relative*	Pairwise Similarity (%)	Length of sequence (bp)
M1: *Anemonia sulcata* (*tentacles*)	M1-5-2	KX369290	*Bacillus subtilis *group	99.93	1432
	M1-33	KX369291	*Bacillus subtilis *group	99.93	1415
M2: *Anemonia sulcata* (gut)	M2-12	KX369292	*Bacillus subtilis *group	99.86	1436
	M2-16-2	KX369293	*Bacillus pumilus *group	98.87	1571
	M2-61	KX369294	*Bacillus pumilus *group	99.93	1425
M3: *Holothuria tubulosa* (intestines)	M3-59	KX369295	*Bacillus subtilis *group	99.67	1520
M4: *Holothuria tubulosa* (coelomic fluid)	M4-71	KX369296	*Bacillus pumilus *group	100.00	1431
M6: *Holothuria forskali* (coelomic fluid)	M6-1	KX369297	*Bacillus subtilis *group	99.93	1405
	M6-12-2	KX369298	*Vibrio alginolyticus *group	99.86	1387
	M6-25	KX369299	*Bacillus subtilis *group	99.93	1348
	M6-26-1	KX369300	*Vibrio alginolyticus *group	99.86	1405
	M6-26-2	KX369301	*Vibrio alginolyticus group*	99.85	1379
	M6-33	KX369302	*Pseudoalteromonas tetraodonis *group	100.00	958
	M6-45	KX369303	*Bacillus subtilis *group	99.90	978
M7: *Actinia equina (tentacles)*	M7-11-1	KX369304	*Vibrio alginolyticus *group	99.86	1412
	M7-11-2	KX369305	*Pseudoalteromonas tetraodonis *group	99.50	1409
M8: *Actinia equina (gut)*	M8-1	KX369306	*Bacillus subtilis *group	100.00	1398
	M8-2	KX369307	*Bacillus subtilis *group	100.00	1194
	M8-6	KX369308	*Bacillus subtilis *group	100.00	1395
	M8-15	KX369309	*Bacillus marisflavi* TF-11^T^	100.00	1360
	M8-24-1	KX369310	*Vibrio alginolyticus *group	99.86	1429
M9: *H*. *tubulosa* and *H*. *forskali* (feces)	M9-11	KX369311	*Bacillus subtilis *group	100.00	1390
	M9-27-1	KX369312	*Stenotrophomonas maltophilia *group	99.93	1398
	M9-44	KX369313	*Bacillus pumilus *group	99.93	1344
	M9-53-1	KX369314	*Bacillus aerophilus *group	99.93	1392
	M9-53-2	KX369315	*Psychrobacter faecalis *group	99.77	1340
	M9-61	KX369316	*Bacillus anthracis *group	99.93	1375

Most of the phylogenetic affiliations belong to taxonomic groups. A taxonomic group includes species/subspecies that are not distinguishable by their 16S rRNA gene sequences. The definite species, according to EzTaxon [53], included in these taxonomic groups are shown in S2 Table.

### Diversity and relative importance of the isolated bacteria in the holobiont microbiomes

The OTUs in the microbiota of each holobiont were identified using the V4 hypervariable region of 16S rRNA gene. In the Table 4 we show the results of the number of sequences (reads), detected OTUs richness (S), Shannon-Wiener’s diversity index, Pielou’s evenness index, Alpha diversity and OTUs richness estimations (Chao and ACE indexes with standard errors) pooling all the sequences of the same holobiont. *Holothuria tubulosa* was the holobiont with more OTUs (38713) in its microbiome followed by *H*. *forskali* (15898).

**Table 4:** Number of sequences, OTUs (97% sequence similarity), and richness, and diversity indexes estimated for the microbiome of each holobiont, holothurian feces, and seawater collected from the aquaculture tank.

Samples	Total number of sequences (reads)	Detected richness OTUs (S)	Diversity (Shannon–Wiener, H)	Species evenness (Pielou’s index, J)	Alpha diversity (α)	Estimated Richness (S Chao ± se Chao)	Estimated Richness (S ACE ± se ACE)
*Anemonia sulcata* (SamplesM1+M2)	58839	5483	4.94	0.57	1478	10887 ± 272	11750 ± 71
*Actinia equina* (Samples M7 + M8)	4065	254	2.99	0.54	60	630 ± 87	821 ± 20
*Holothuria forskali* (Samples M5+M6)	375177	15898	4.35	0.45	3366	54867 ± 1240	54898 ± 52
*Holothuria tubulosa* (Samples M3+M4)	392169	38713	6.60	0.62	10658	63609 ± 475	70507 ± 171
Holothurian feces (Sample M9)	10838	462	2.37	0.39	98	2109 ± 330	1896 ± 27
Seawater (Sample M10)	37215	2053	4.49	0.59	468	4596 ±219	4616 ± 43

The microbiomes of *Holothuria tubulosa* and *Holothuria forskali* showed the highest estimates of OTU richness, followed by *Anemonia sulcata*. *Holothuria tubulosa* showed the highest diversity (6.60), followed by *Anemonia sulcata* (4.94) according to Shannon-Wiener diversity index. In the case of alpha diversity, *Holothuria tubulosa* (10658), *Holothuria forskali* (3366) and *Anemonia sulcata* (1478) showed the highest indexes. Holothurian feces presented low diversity compared to the other samples. Pielou’s index varied from 0.39 in holothurian feces to 0.62 in *Holothuria tubulosa*. We also obtained the rarefaction curves for the microbiome of each holobiont (S4 and S5 Figs).

**Fig 1:**
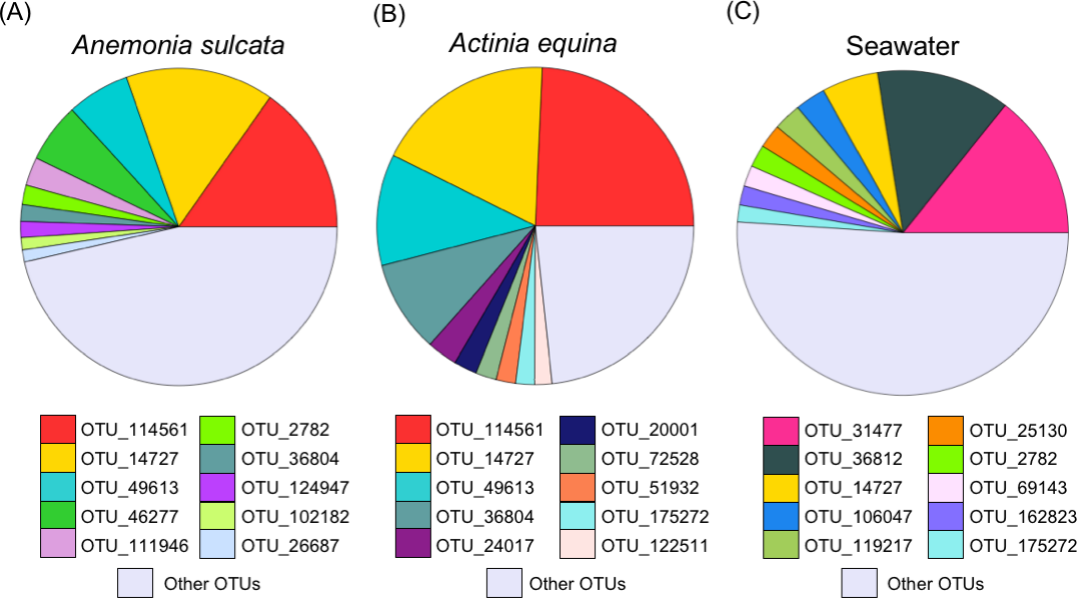
Relative contribution of the first ten operational taxonomical units (OTUs) in the microbiome of sea anemones and in the seawater of the aquaculture tank. OTUs relative contribution in the microbiome of (A) *Anemonia sulcata*, (B) *Actinia equina*, and (C) seawater in the tank. OTU taxon assignments are shown in S3 Table. Note “other OTUs” contribution includes different taxa for each specific holobiont and seawater.

**Fig 2:**
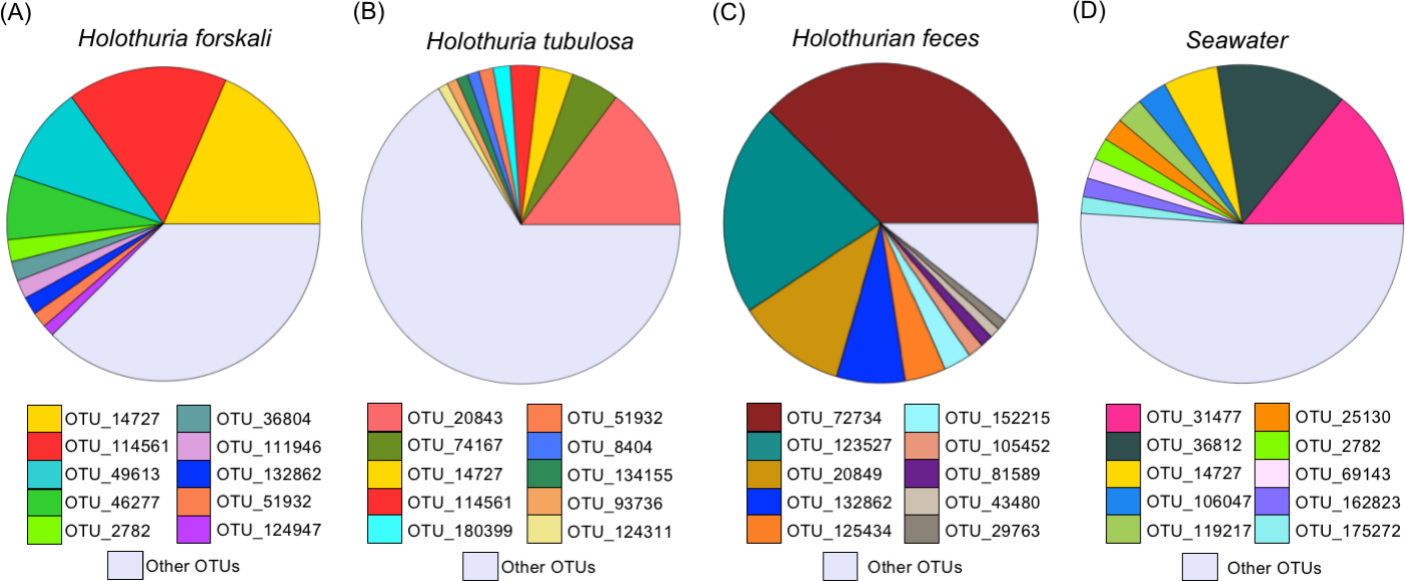
Relative contribution of the first ten operational taxonomical units (OTUs) in the microbiome of holothurians, in feces and in the seawater of the aquaculture tank. OTUs relative contribution in the microbiome of (A) *Holothuria forskali*, (B) *Holothuria tubulosa*, (C) holothurian feces, and (D) seawater in the tank. OTU taxon assignments are shown in S3 Table. Note “other OTUs” contribution includes different taxa for specific each holobionts and seawater.

The taxonomical identification of the ten more frequent OTUs for each microbiome is shown in the S3 Table. The two most frequent OTUs in the sea anemones (OTU 114561 and OTU 14727) were similar (Fig 1A y 1B) and differed substantially of the bacteria found in the seawater from the aquaculture tank (Fig 1C). The most common OTU belonged to the phyllum *Bacteroidetes*, order *Flavobacteriales* and family *Flavobacteriaceae* (S3 Table). In contrast, the microbiome OTUs composition of the two holothurian species was different between them and also in comparison to the bacteria found in the feces and in the seawater from the aquaculture tank (Fig 2). Interestingly, *Holothuria forskali* (Fig 2A) showed a high similarity in OTUs composition with the sea anemones (Fig 1A and 1B). In the case of *H*. *tubulosa* (Fig 2B), besides *Flavobacteriaceae*, other groups as order *Bacteroidales* (phyllum *Bacteroidetes*) and family *Flammeovirgaceae* (phyllum *Bacteroidetes*, order *Cytophagales*) were found. The OTUs found in holothutian feces (Fig 2C) were very different (Fig 2), although the phyllum *Bacteroidetes* also showed a high frequency, along with the phyllum *Actinobacteria* (orden *Actinomycetales*). In the seawater from the tank (Fig 1C and Fig 2D), most OTUs were different of holobiont microbiomes.

**Fig 3:**
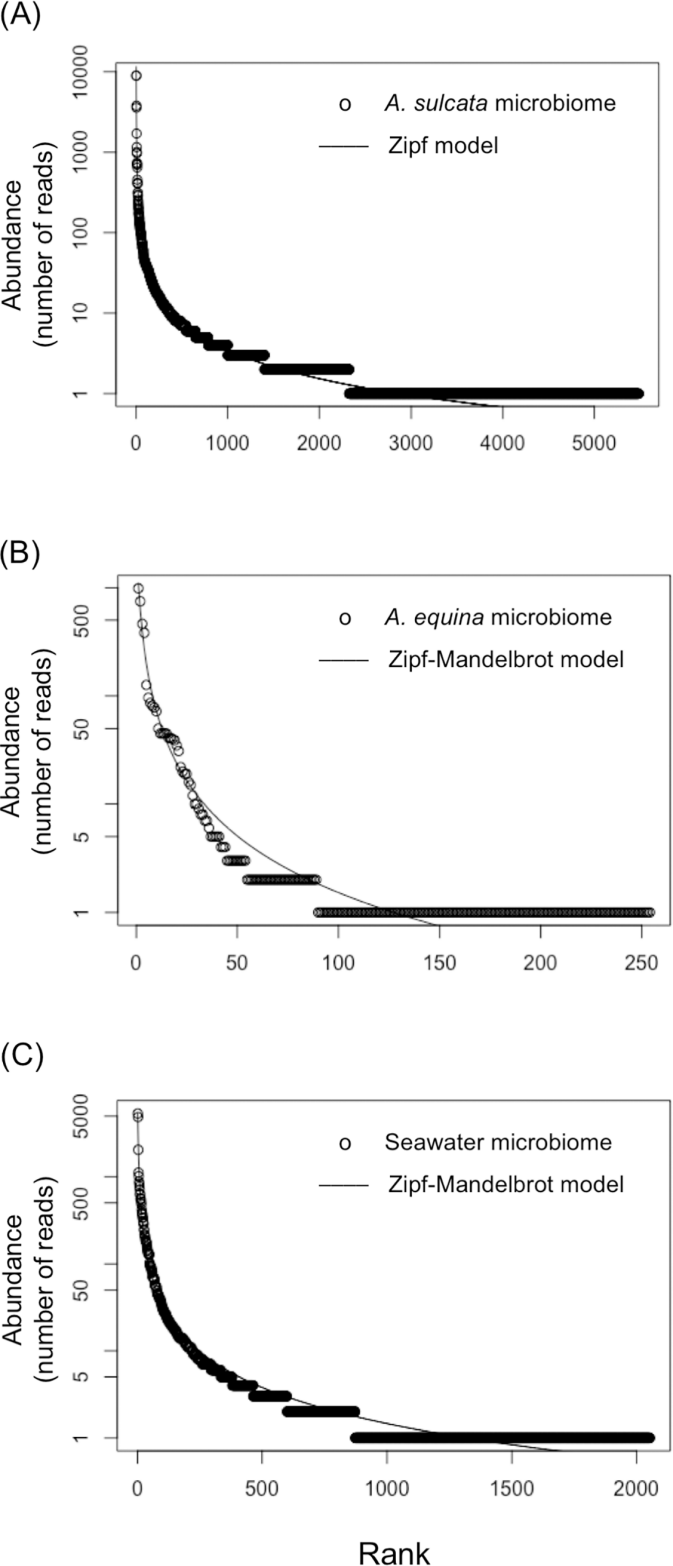
Rank Abundance Dominance (RAD) plots of the sea anemone microbiomes and seawater and the model with the best fit. RAD plots of the microbiome of (A) *Anemonia sulcata*, (B) *Actinia equina*, and (C) seawater of the tank.

**Fig 4:**
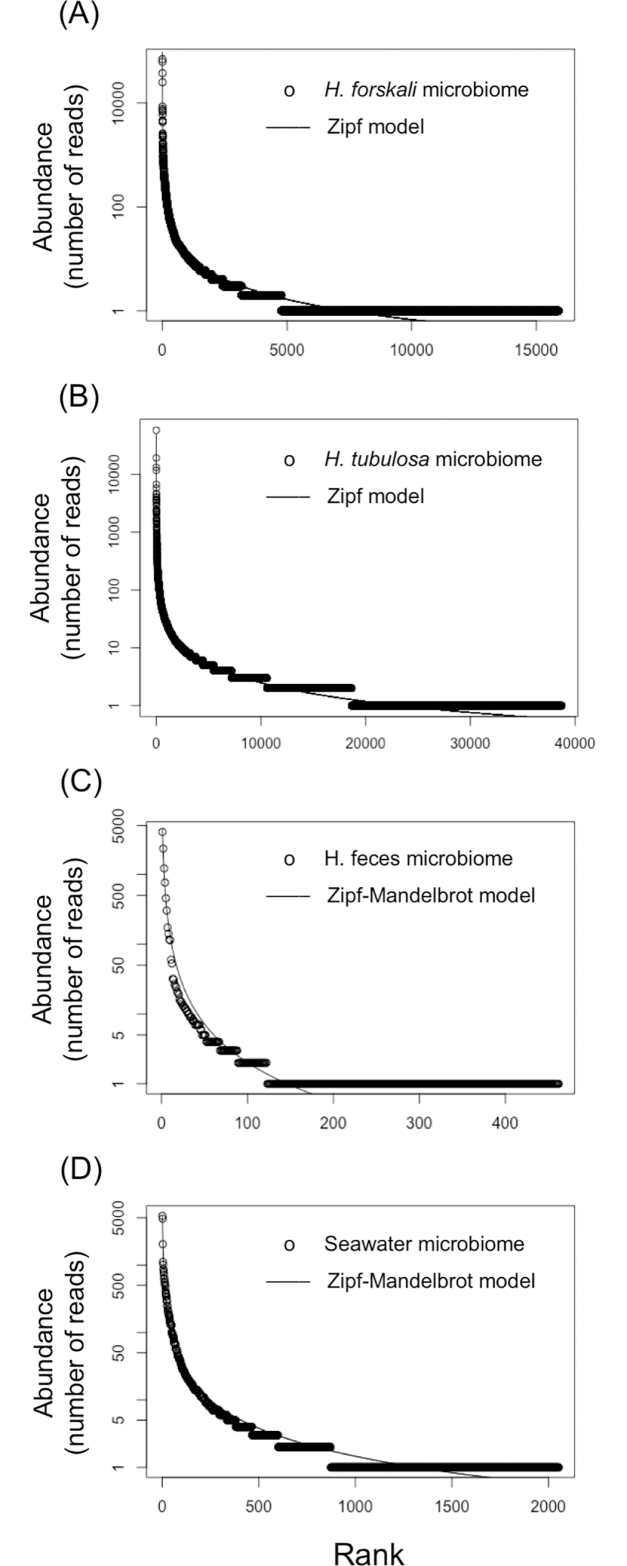
Rank Abundance Dominance (RAD) plots of the holothurians, feces and seawater and the model with the best fit. RAD plots of the microbiome of (A) *Holothuria forskali*, (B) *Holothuria tubulosa*, (C) holothurian feces, and (D) seawater of the tank.

To determine the rank-abundance dominance (RAD) of the sequences among the different OTUs in the microbiome of each holobiont we tested five models: null (brokenstick), preemption, lognormal, zipf and zipf-mandelbrot (S4 Table). The best fit was the model with the lower AIC value (Figs 3 and 4). All the curves for the different models are shown in S6 and S7 Figs. The RAD of the microbiome of *A*. *sulcata* (Fig 3A) and of the two holothurians fitted better to the Zipf model, whereas for the rest of samples the best fits were to the Zipf- Mandelbrot model (Fig 4A and 4B).

We determined the relative abundance of the isolated bacteria with antimicrobial activity by tracking their specific V4 sequences in the total pool of V4 sequences obtained in the massive sequencing for each sample of the sea anemones and holothurians (i.e. we tracked, for instance, the V4 sequences of the isolated bacteria with antimicrobial activity from the M6 sample with all the V4 sequences found in the massive sequencing of the microbiome from the coelomic fluid of *Holothuria forskali*). We found coincidences at 100% of similarity only for the M6 isolated strains: M6-12-2 (*Vibrio alginolyticus* group), M6-26-1 (*V*. *alginolyticus* group) and M6-26-2 (*V*. *alginolyticus* group) that appeared with the frequency of 0.0032%. We found more coincidences but using at lower levels of similarity (Table 5). For instance, at a 98% of similarity the *V*. *alginolyticus* group increased its frequency one order of magnitude up to 0.0141% in the *Holothuria forskali* microbiome. Bacterial coincidence also appeared with 99.6% similarity to M6-33 (*Pseudomonas tetraodonis* group) with a frecuency of 0.0005% in the *H*. *forskali* microbiome.

**Table 5:** Relative abundance (%) of bacteria with antimicrobial potential in the whole microbiome of the study holobionts using different similarity thresholds.

Strain ID	Identification	Similarity thresholds (%)					
		100.00	99.60	99.20	98.80	98.40	98.00
M6-12-2	*Vibrio alginolyticus*	0.0032	0.0093	0.0115	0.0128	0.0133	0.0141
M6-22-2	*Vibrio alginolyticus*	0.0032	0.0093	0.0115	0.0128	0.0133	0.0141
M6-26-1	*Vibrio alginolyticus*	0.0032	0.0093	0.0115	0.0128	0.0133	0.0141
M6-26-2	*Vibrio alginolyticus*	0.0032	0.0093	0.0115	0.0128	0.0133	0.0141
M6-33	*Pseudomonas tetraodonis*	0.0000	0.0005	0.0005	0.0005	0.0013	0.0019

## Discussion

In the holobionts *Anemonia sulcata*, *Actinia equina*, *Holothuria forskali*, and *H*. *tubulosa* we found symbiotic bacteria with antibacterial activity against human, plants or aquaculture pathogens (Table 1) and antifungal activity against plant pathogens (Table 2). The results of the antimicrobial screenings suggest an important biotechnological potential of the microbiota of these marine invertebrates. These results are consistent with previous studies. For instance, the culture broth of *Bacillus amyloliquefaciens* SCSIO 00856 isolated from sea gorgonian had strong antibacterial activity against *Escherichia coli*, *Bacillus subtilis*, and *Staphyloccocus aureus* [27]. In the black coral *Antipathes dichotoma*, the 51.6% of the microbial isolates exhibited significant antibacterial and antifungal activities [31]. Similarly, bacteria associated with the soft coral *Sarcophyton glaucum* showed antimicrobial activity against different pathogens [33]. Antimicrobial peptides have been also detected in the coelomic fluid of sea cucumber species [35,40,65]. Antifungal compounds as triterpene glycosides have been also found in holothurian extracts and these compounds have activity against human [38] and plant pathogens [34,37,66].

In the isolated strains with antimicrobial potential, we found a predominance of the genus *Bacillus* (Table 3). This genus is a prolific source of bioactive compounds with antimicrobial, antiviral, immunosuppressive, and antitumor activity [67,68]. New *Bacillus* species with antibacterial activity have been isolated from Malaysian sea cucumbers [41]. In the bacterium *Bacillus amyloliquefaciens* SCSIO 00856, isolated from the gorgonian *Junceella juncea*, several antibacterial compounds have been detected [27]. This last bacterium was also isolated in our study. Antifungal substances produced by the marine *Bacillus* sp. 109GGC020 against the phytopathogen fungus *Phytophthora capsici* have been also found in previous studies [69]. In fact, several authors have remarked that marine microorganisms can be an promising alternative in the search of antimicrobial substances [70–72]. These microorganisms are highly diversified and may have unique metabolic pathways, producing metabolites that would not be discovered in the terrestrial realm.

To study the microbiota associated with each holobiont we identified the bacterial OTUs using their V4 hypervariable region of 16S rRNA gene and determined its diversity and rank-abundance dominance (RAD) in each microbiome. The diversity of *H*. *tubulosa* microbiome was the highest value and was similar to values previously reported for the gut of the holothurian *Apostichopus japonicus* [73]. However, the *H*. *forskali* microbiome had a lower diversity despite the number of total sequences retrieved was similar. Holothurian feces showed low richness and diversity compared with the two holothurian microbiomes. The best fits of RAD were obtained for the Zipf and Zipf-Mandelbrot models (Figs 3 and 4, S4 Table). Long tails of species with low number of representatives characterize these models. Zipf and the Zipf-Mandelbrot models are similar and Zipf model can be considered as a subset of Zipf-Mandelbrot [74]. These two models are based on the information that is accumulated in the system and the information costs [75]. That is, the presence of a species depends on the preceding physical conditions and on the previous presence of species (named costs). Pioneer species have low costs, requiring few preceding conditions, whereas late successional species have higher costs before they can occupy the niche and, consequently, they will be rare species [74,76]. Zipf-Mandelbrot model can be interpreted as many factors acting sequentially during the species establishment [74], whereas Log-normal model, for instance, can be interpreted as the result of many factors interacting simultaneously on species establishment. The colonization and establishment of symbiotic bacteria in the holobiont is likely a complex and sequential process where pioneer bacteria might have advantage over the last ones.

The bacterial composition of the microbiome of *Anemonia sulcata*, *Actinia equina*, *Holothuria tubulosa* and *H*. *forskali* showed a predominance of the phyllum *Bacteroidetes* (family *Flavobacteriaceae*). Previous studies, using different techniques, have reported different microbiome composition in other species of *Cnidaria*. For instance, the predominant bacteria in *Anemonia viridis* belonged to *Firmicutes* [28] and in the zoanthid *Palythoa australiae* to *Proteobacteria* [77]. In sea cucumbers, as *Apostichopus japonicus*, *Proteobacteria* was also the predominant phylum in its gut content [73] and phylogenetically unique members of *Epsilonproteobacteria* and *Alphaproteobacteria* (order *Rickettsiales*) have been discovered [40]. These differences could be related to different life conditions of the holobionts. The above-mentioned studies used individuals from natural environments, whereas we used individuals from an aquaculture tank. The growth and life-style in an aquaculture tank could cause nutritional changes and different bacterial composition during the consolidation process of holobiont microbiotas. More studies are needed to demonstrate or not the plasticity level of symbiotic bacteria in wild vs. captive conditions. The microbiomes of these two sea anemones were similar between them but very different of the bacterial composition in the aquaculture tank. This fact suggests a selective process during colonization and consolidation of the microbiota. On the other hand, the two holothurian microbiomes were very different between them and different of the seawater bacteria composition in the aquaculture tank. The colonization by symbiontic bacteria appears to be highly selective and restrictive process similar to a bottleneck effect [78]. Maynard et al. [79] described the process with many physical and chemical interactions in sequential steps. It is also remarkable that the bacterial composition in the microbiome of *Holothuria forskali* was more similar to the microbiomes of the two sea anemones than to the microbiome of *Holothuria tubulosa*, despite their close phylogeny. A plausible explanation could be based on the differences in the ecological niches of both holothurian species. In our study, the sea cucumbers were collected from the same tank, but in the natural environment they have completely different niches. *Holothuria tubulosa* Gmelin, 1788 is usually found on sandy seabeds, among seagrass and on muddy rocks [80,81]. In contrast, *Holothuria forskali* Delle Chiaje 1823 is found on detrital seabeds, on rocks or stones, with a preference for vertical faces [80,81] coexisting with *Anemonia sulcata* Pennant, 1766 and *Actinia equina mediterranea* Schmidt, 1971 [80]. We hypothesized that holobionts living in similar niches could share symbiotic bacteria irrespectively, to some extent, of their phylogenies. A similar conclusion was obtained in microbiomes of marine sponges [46]. In this last study, symbiotic bacteria in sponges from distant locations and phylogenies around the world were analyzed. The authors did not find a significant relationship between the similarity of symbiotic bacteria and the host phylogeny. They concluded that symbiotic bacteria are probably acquired via environmental transmission. In our study, symbiotic bacteria in the different holobionts did not suggest a strong dependence of host phylogeny at least for the case of the two species of holothurians.

Insights into the prevalence of the bacteria with antimicrobial activity in the whole microbiome of the anemones and holothurians studied can be relevant to determine their biotechnological potentiality. We tracked coincidences (from a 98.00 to a 100% of similarity) between the V4 hypervariable region in the whole pool of sequences obtained with the Illumina platform and the V4 hypervariable region of the bacteria with antimicrobial activity. We only found coincidences only in the case of the microbiome of *Holothuria forskali*, although at a very low frequency (Table 5). This result suggests that these symbiotic bacteria are OTUs relatively rare in their microbiomes. The most plausible reason why we did not find the sequences of the isolated bacteria was the extremely long tails in their RAD (Figs 3 and 4) implying an extraordinary difficulty to detect them. This result is not surprising since the RAD curves with the best fits were, precisely, the most skewed. In general, bacteria abundance-rank distribution in marine environment contain many rare species and just a few common (core) species [82]. Indeed, these skewed curves have been observed both in natural marine ecosystems and in holobiont microbiomes [42,43,45,46]. Likely, the core taxa are responsible for carbon and energy flow and basic functions inside the holobionts, whereas the rare taxa, which survive at low abundance, represent a seed bank of genetic diversity and antimicrobial potential to face eventual pathogen outbreaks [43,45,46].

In this study, we were able to isolate symbiotic bacteria that produce antimicrobial substances against human, plants or aquaculture pathogens from marine invertebrate holobionts. These bacteria were hardly detected with massive MiSeq Illumina sequencing, but we were able to culture them despite their low relative abundances. Therefore, we still need pure classical culture and new approaches as cocktail and miniaturized cultures and nature incubators [9] to improve our knowledge on the great biotechnological capacities of symbiotic bacteria in marine invertebrate holobionts since they appear to be constituent of the rare biosphere.

## Supporting information

S1 Fig Antibacterial pre-screening plate using microtiter replicator over 135 mm diameter plate. Antibacterial screening plate against *Staphylococcus aureus* showing two small inhibition halos (B11, C10) and one big inhibition halo (A6, A7, A8, B6, B7).
(JPG)

S2 Fig Antibacterial screening plate using the well diffusion agar-plate technique and stainless steel cylinders. Antibacterial screening against *Staphylococcus aureus* showing the inhibition halo of strain M8-15 (30 mm).
(JPG)

S3 Fig Antifungal screening in 48-well microtiter plate. Antifungal screening plate against *Phytophthora cinnamomi* showing the inhibition activity of strains M7-06 (C1), M8-2 (C3), M8-13 (D1), M9-33 (E3), M9-44 (E5). The negative control was fungal culture with cycloheximide (50 mg/mL) (F4).
(JPG)

S4 Fig **Rarefaction curve using Illumina reads for the *Anemonia sulcata* (A), *Actinia equina* (B) and seawater (C) microbiomes**.
(TIFF)

S5 Fig **Rarefaction curve using Illumina reads for the *Holothuria forskali* (A), *Holothuria tubulosa* (B), holothurian feces (C) and seawater (D) microbiomes**.
(TIFF)

S6 Fig **OTU abundance distribution indicating the five fitted models (Null, Preemption, Lognormal, Zipf and Zipf-Mandelbrot) and relative abundance (%) rank distribution for the *Anemonia sulcata* (A, D), *Actinia equina* (B, E) and seawater (C, F) microbiomes**.
(TIFF)

S7 Fig **OTU abundance distribution indicating the five fitted models (Null, Preemption, Lognormal, Zipf and Zipf-Mandelbrot) and relative abundance (%) rank distribution for the *Holothuria forskali* (A, E), *Holothuria tubulosa* (B, F), holothurian feces (C, G) and seawater (D, H) microbiomes**.
(TIFF)

S1 Table Inhibition halo diameter (mm) measured after the antibacterial screening against the eight indicator bacteria. (DOCX)

S2 Table Species included in each taxonomic group according to EzTaxon [53]. (DOCX)

S3 Table Relative contribution (%) and taxonomical identification of the ten most frequent OTUs in the microbiomes of sea anemones, holothurians, feces and seawater from the aquaculture tank. (DOCX)

S4 Table Akaine’s Information Criterion (AIC) values for the five fitted models (null, preemption, lognormal, zipf and zipf-mandelbrot) for each sample. (DOCX)
