# Supplementary figures and images for: Diversity and antimicrobial potential in sea anemone and holothurian microbiomes
Source: PLoS One. 2018 May 9;13(5):e0196178. doi: 10.1371/journal.pone.0196178 (PMC5942802; doi:10.1371/journal.pone.0196178)

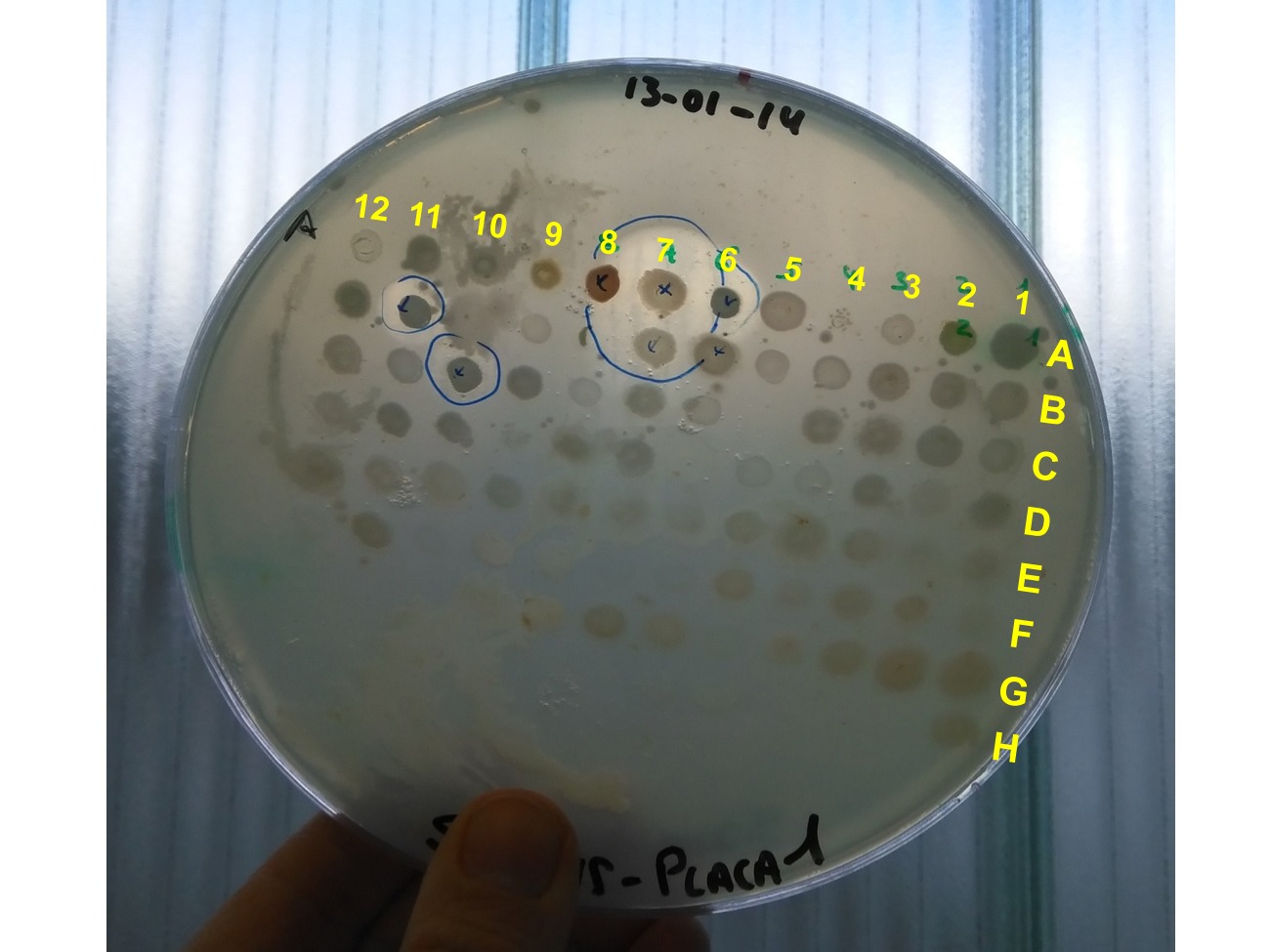

Supplement: S1 Fig — Antibacterial screening plate against Staphylococcus aureus showing two small inhibition halos (B11, C10) and one big inhibition halo (A6, A7, A8, B6, B7). (JPG) [file pone.0196178.s001.jpg]

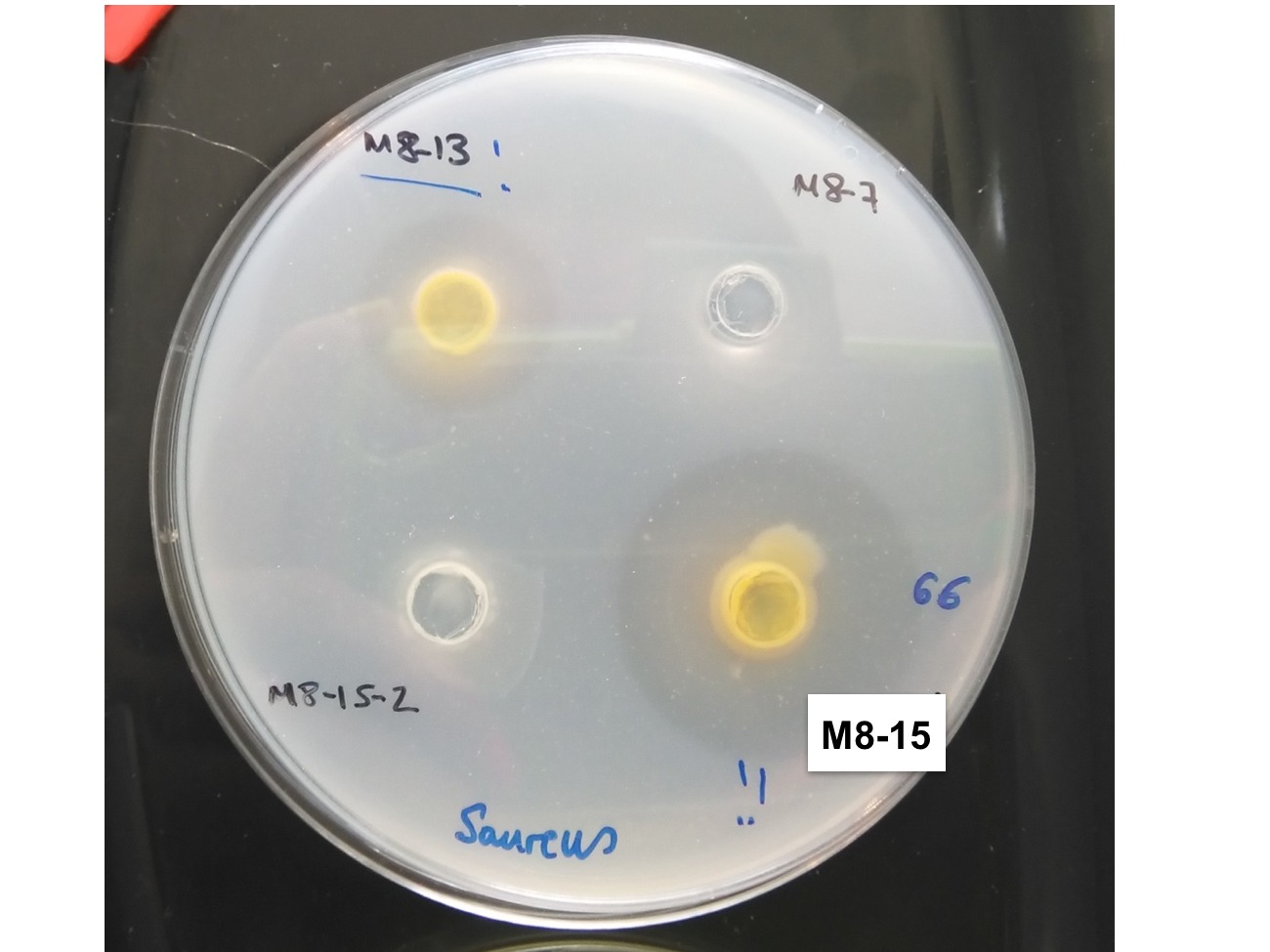

Supplement: S2 Fig — Antibacterial screening against Staphylococcus aureus showing the inhibition halo of strain M8-15 (30 mm). (JPG) [file pone.0196178.s002.jpg]

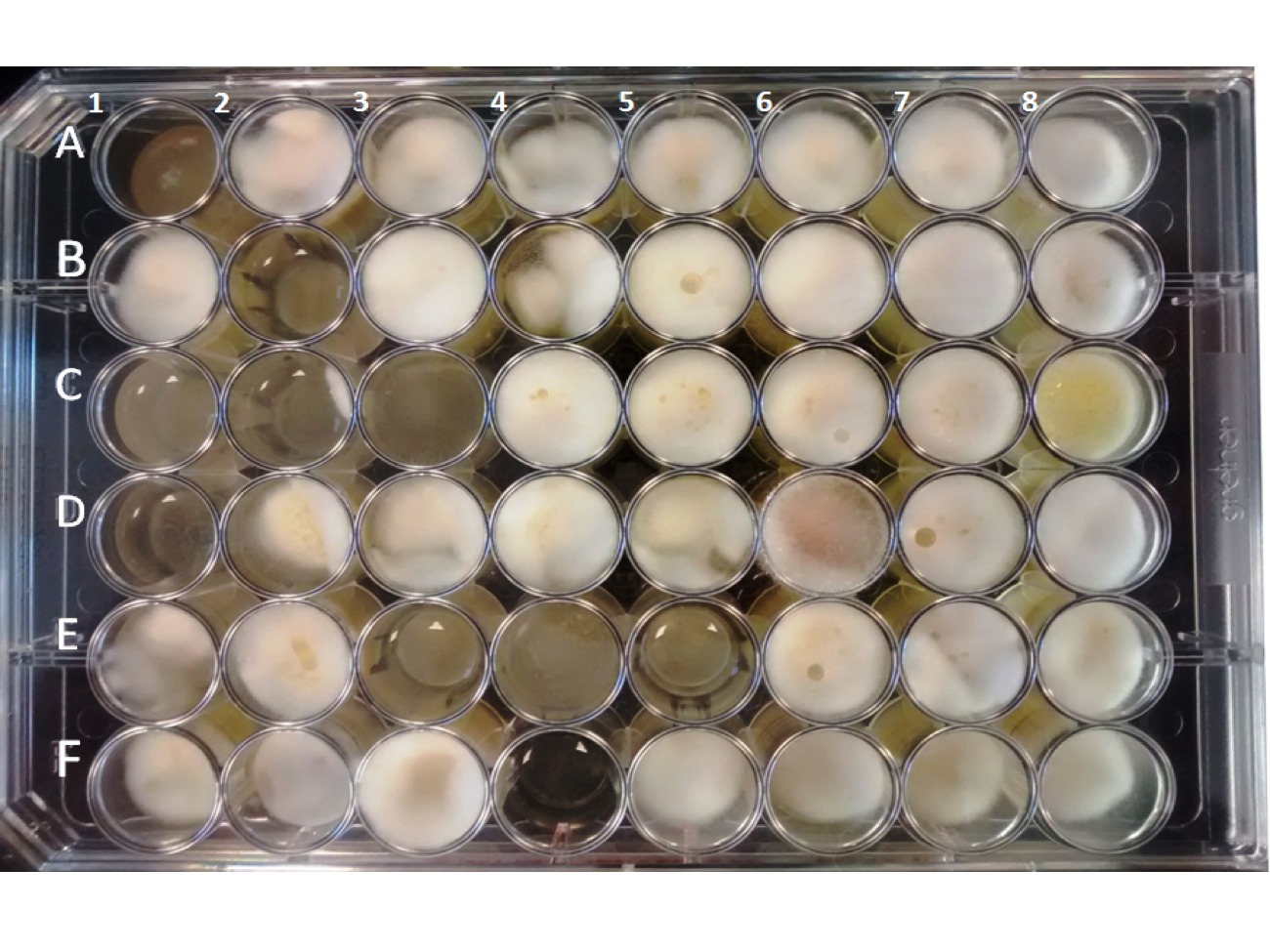

Supplement: S3 Fig — Antifungal screening plate against Phytophthora cinnamomi showing the inhibition activity of strains M7-06 (C1), M8-2 (C3), M8-13 (D1), M9-33 (E3), M9-44 (E5). The negative control was fungal culture with cycloheximide (50 mg/mL) (F4). (JPG) [file pone.0196178.s003.jpg]

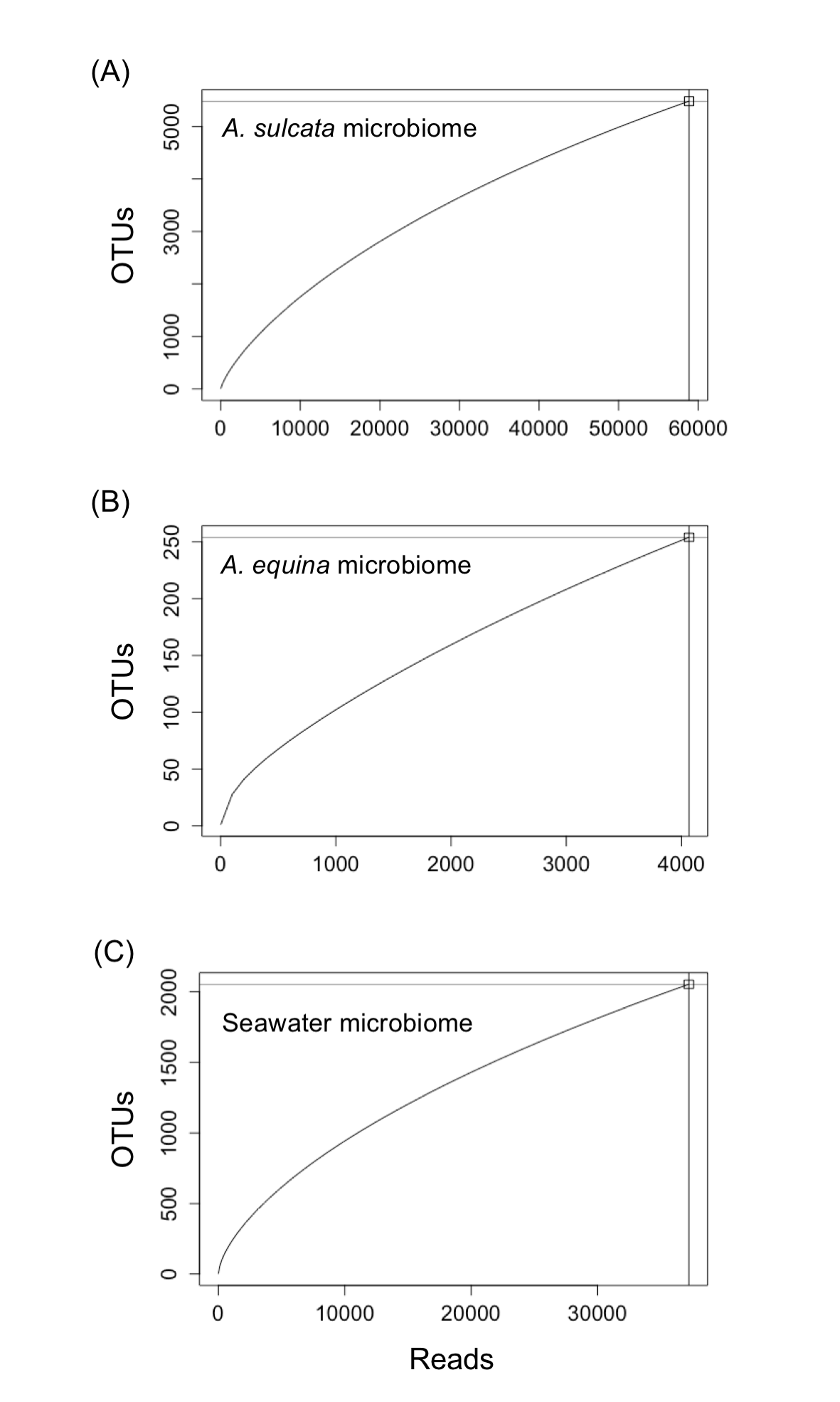

Supplement: S4 Fig — Rarefaction curve using Illumina reads for the Anemonia sulcata (A), Actinia equina (B) and seawater (C) microbiomes. (TIFF) [file pone.0196178.s004.tiff]

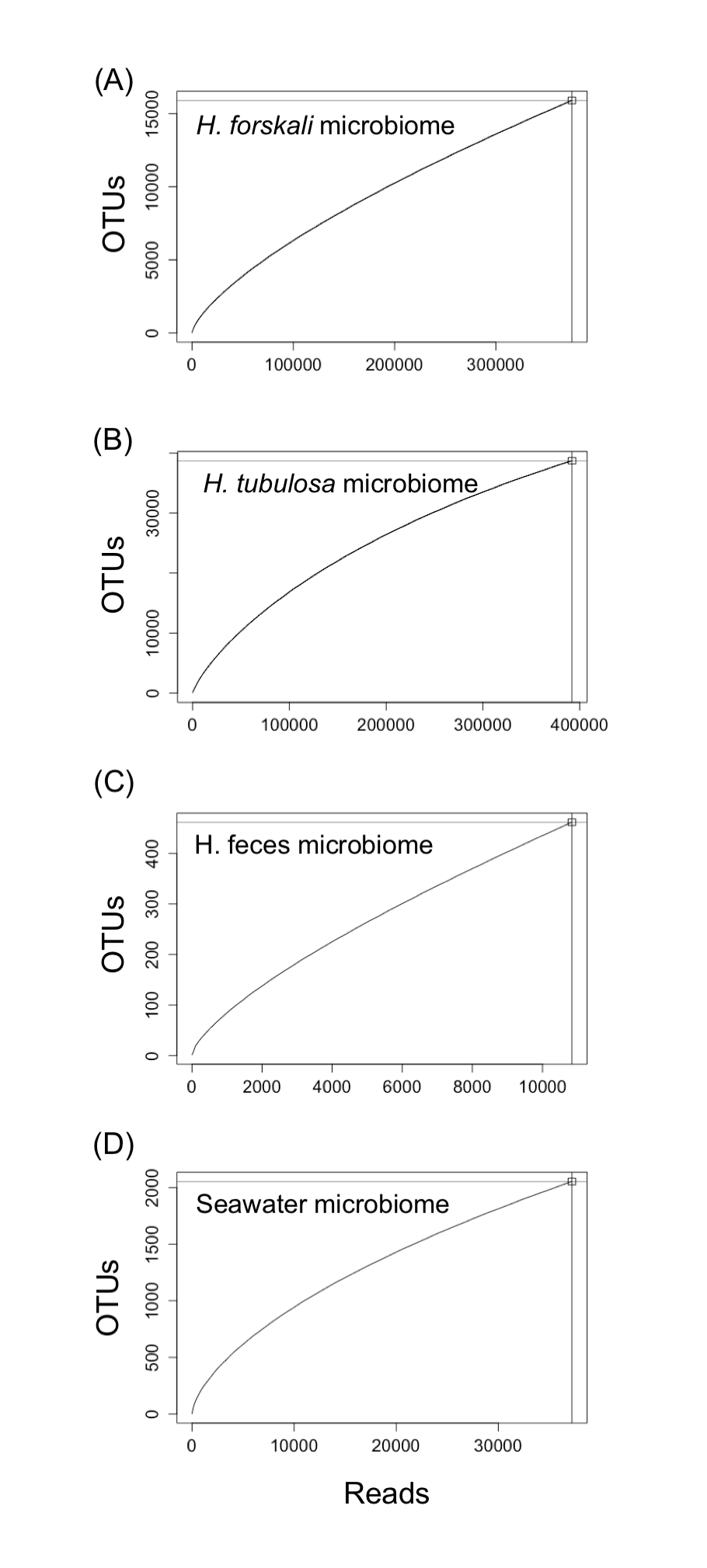

Supplement: S5 Fig — Rarefaction curve using Illumina reads for the Holothuria forskali (A), Holothuria tubulosa (B), holothurian feces (C) and seawater (D) microbiomes. (TIFF) [file pone.0196178.s005.tiff]

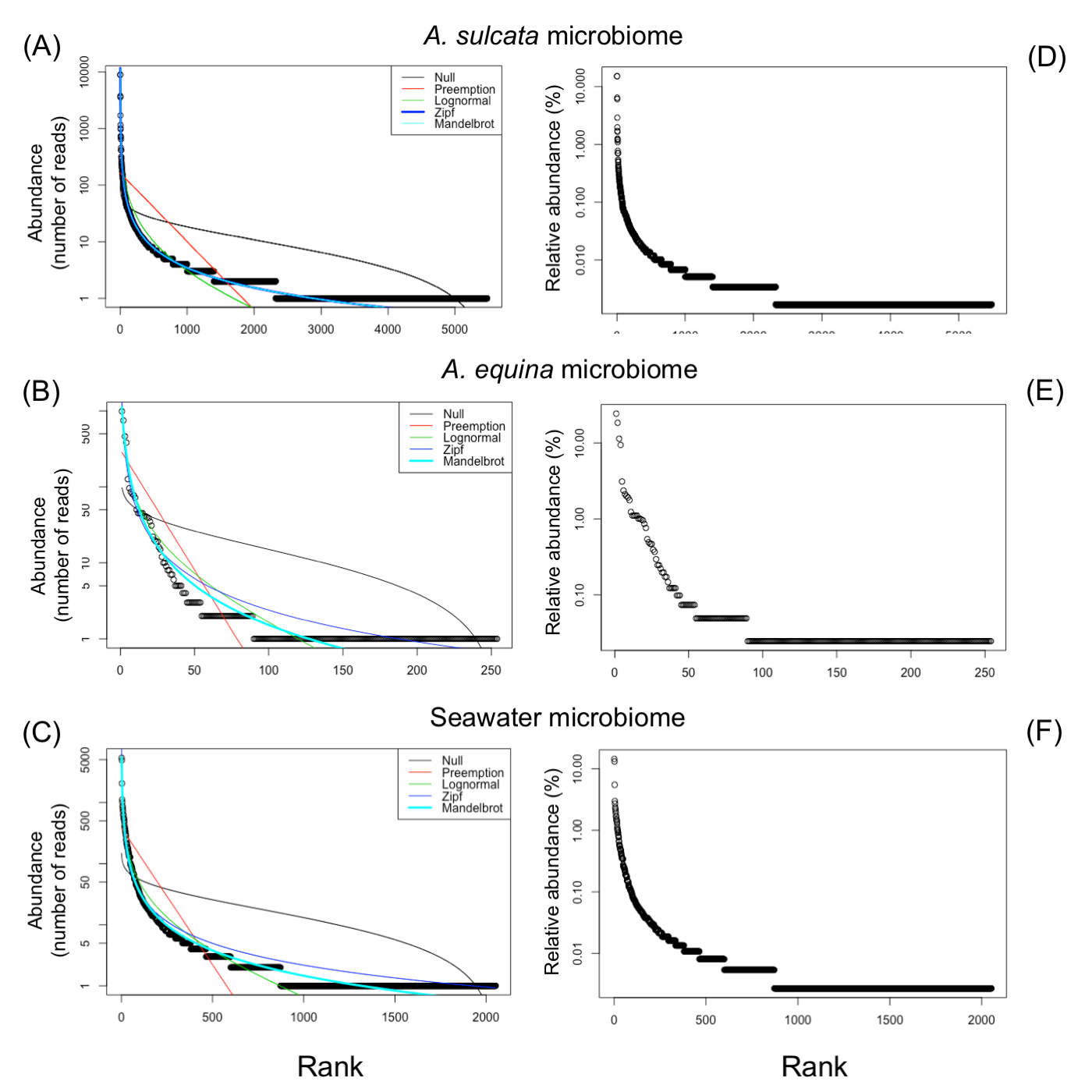

Supplement: S6 Fig — OTU abundance distribution indicating the five fitted models (Null, Preemption, Lognormal, Zipf and Zipf-Mandelbrot) and relative abundance (%) rank distribution for the Anemonia sulcata (A, D), Actinia equina (B, E) and seawater (C, F) microbiomes. (TIFF) [file pone.0196178.s006.tiff]

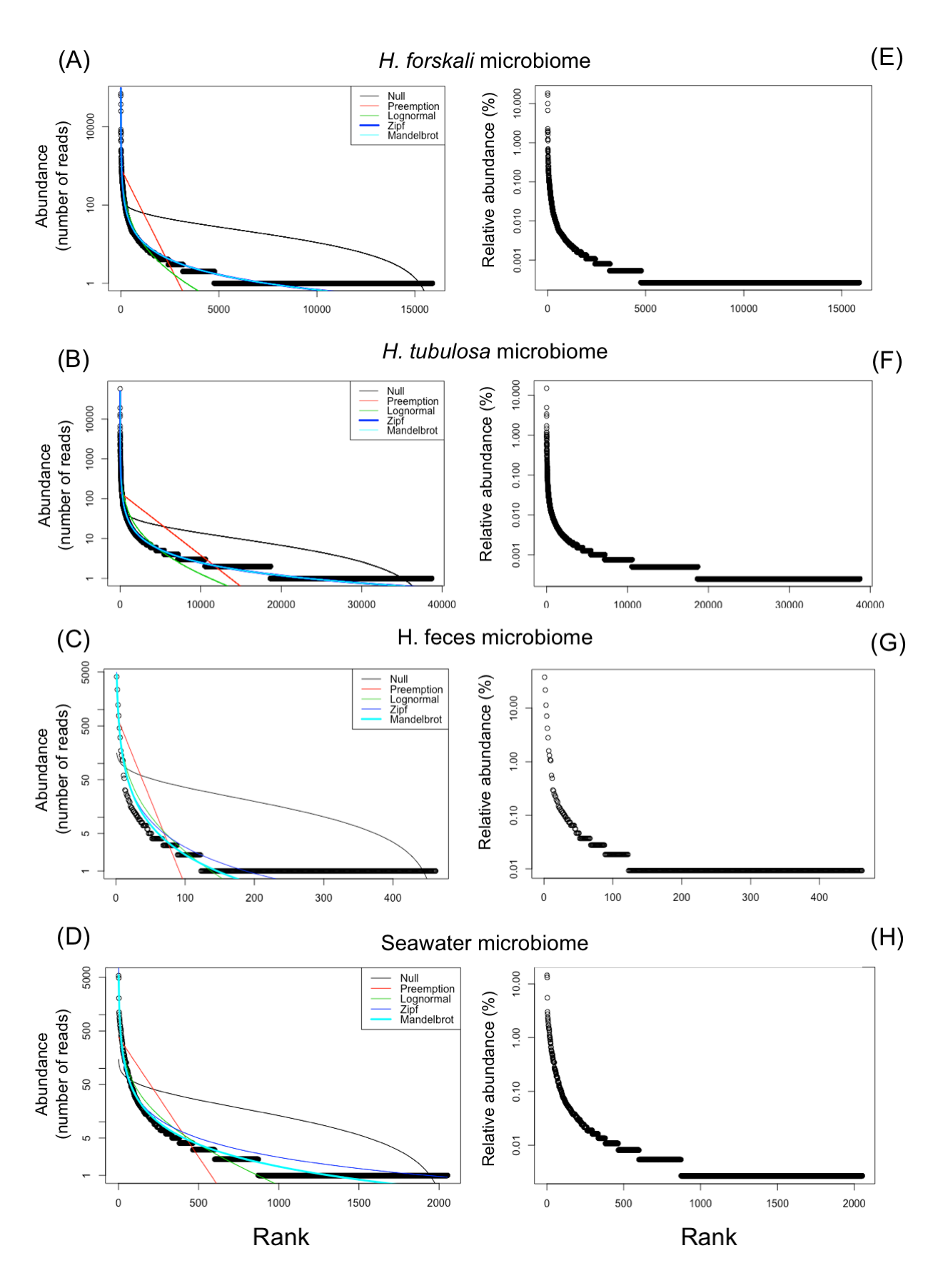

Supplement: S7 Fig — OTU abundance distribution indicating the five fitted models (Null, Preemption, Lognormal, Zipf and Zipf-Mandelbrot) and relative abundance (%) rank distribution for the Holothuria forskali (A, E), Holothuria tubulosa (B, F), holothurian feces (C, G) and seawater (D, H) microbiomes. (TIFF) [file pone.0196178.s007.tiff]
