# Supplementary material for: Diversity and antimicrobial potential in sea anemone and holothurian microbiomes
Source: PLoS One. 2018 May 9;13(5):e0196178. doi: 10.1371/journal.pone.0196178 (PMC5942802; doi:10.1371/journal.pone.0196178)
Supplement: S1 Table — (DOCX) [file pone.0196178.s008.docx]

| Sample | Isolate ID | *Staphylococcus aureus* | *Erwinia amylovora* | *Pseudomonas aeruginosa PAO1* | *Agrobacterium tumefaciens* | *Vibrio brasiliensis* | *Vibrio anguillarum* | *Vibrio mediterranei* | *Vibrio coralliilyticus* |
| --- | --- | --- | --- | --- | --- | --- | --- | --- | --- |
| M1: *Anemonia sulcata* (tentacles) | M1-5-2 | 15 | - | - | - | - | 13 | - | - |
|  | M1-33 | 13 | 15 | - | - | - | - | - | - |
| M2: *Anemonia sulcata* (gut) | M2-12 | 14 | 15 | - | - | - | - | - | - |
|  | M2-16-2 | 16 | 15 | - | - | - | 17 | 17 | - |
|  | M2-61 | 16 | 22 | - | - | - | 21 | 17 | - |
| M3:  *Holothuria tubulosa* (intestines) | M3-59 | 14 | - | - | 20 | - | - | - | - |
| M4:  *Holothuria tubulosa* (coelomic fluid) | M4-71 | 20 | - | - | - | - | 16 | - | - |
| M6: *Holothuria forskali* (coelomic fluid) | M6-1 | - | 18 | - | - | - | - | - | - |
|  | M6-12-2 | 14 | - | 12 | - | - | - | - | - |
|  | M6-25 | 13 | - | - | - | - | - | - | - |
|  | M6-26-1 | - | 15 | - | - | - | - | - | - |
|  | M6-26-2 | - | 20 | - | - | - | - | - | - |
|  | M6-33 | - | 14 | - | 22 | - | - | - | - |
|  | M6-45 | 11 | 12 | - | 13 | - | - | - | - |
| M7: *Actinia equina (*tentacles*)* | M7-11-1 | 12 | 23 | - | - | - | - | - | - |
|  | M7-11-2 | 11 | 10 | - | - | - | - | - | - |
| M8: *Actinia equina (*gut*)* | M8-1 | 13 | 14 | - | 26 | - | - | - | - |
|  | M8-2 | 13 | - | - | 16 | - | - | - | - |
|  | M8-6 | 12 | 10 | - | 18 | - | - | - | - |
|  | M8-15 | 30 | - | - | - | - | - | - | - |
|  | M8-24-1 | 11 | 26 | - | - | - | - | - | - |
| M9:  *H. tubulosa* and *H. forskali* (feces) | M9-11 | 13 | 10 | - | 21 | - | - | - | - |
|  | M9-27-1 | - | 34 | - | - | - | - | - | - |
|  | M9-44 | - | 11 | - | - | 15 | 16 | 28 | 18 |
|  | M9-53-1 | 11 | 12 | - | - | - | 14 | 15 | - |
|  | M9-53-2 | - | 10 | - | - | - | - | 15 | - |
|  | M9-61 | - | 11 | - | - | - | 15 | 18 | - |
|  | Total | 19 | 20 | 2 | 7 | 1 | 7 | 6 | 1 |

– no antibacterial activity observed.
