# Supplementary material for: Diversity and antimicrobial potential in sea anemone and holothurian microbiomes
Source: PLoS One. 2018 May 9;13(5):e0196178. doi: 10.1371/journal.pone.0196178 (PMC5942802; doi:10.1371/journal.pone.0196178)
Supplement: S2 Table — (DOCX) [file pone.0196178.s009.docx]

| Taxonomic group | Members |
| --- | --- |
| *Bacillus subtilis* group | *Bacillus subtilis subsp. subtilis*  *Bacillus subtilis subsp. spizizenii*  *Bacillus subtilis subsp. inaquosorum*  *Bacillus mojavensis*  *Brevibacterium halotolerans*  *Bacillus tequilensis*  *Bacillus methylotrophicus*  *Bacillus amyloliquefaciens subsp. amyloliquefaciens*  *Bacillus amyloliquefaciens subsp. plantarum*  *Bacillus siamensis*  *Bacillus vallismortis* |
| *Bacillus pumilus* group | *Bacillus pumilus*  *Bacillus safensis*  *JOTP_s* |
| *Bacillus aerophilus* group | *Bacillus aerophilus*  *Bacillus stratosphericus*  *Bacillus altitudinis*  *Bacillus xiamenensis* |
| *Bacillus anthracis* group | *Bacillus anthracis*  *Bacillus cereus*  *Bacillus thuringiensis*  *Bacillus toyonensis* |
| *Vibrio alginolyticus* group | *Vibrio alginolyticus*  *Vibrio neocaledonicus* |
| *Pseudoalteromonas tetraodonis* group | *Pseudoalteromonas tetraodonis*  *Pseudoalteromonas issachenkonii* |
| *Stenotrophomonas maltophilia* | *Stenotrophomonas maltophilia*  *Stenotrophomonas pavanii* |
| *Psychrobacter faecalis* group | *Psychrobacter faecalis*  *Psychrobacter pulmonis* |
