# Supplementary material for: Diversity and antimicrobial potential in sea anemone and holothurian microbiomes
Source: PLoS One. 2018 May 9;13(5):e0196178. doi: 10.1371/journal.pone.0196178 (PMC5942802; doi:10.1371/journal.pone.0196178)
Supplement: S3 Table — (DOCX) [file pone.0196178.s010.docx]

| Sample | OTU ID | Frecuency (%) | Taxonomical identification |
| --- | --- | --- | --- |
| *Anemonia sulcata* | OTU_114561 | 15.22 | k__Bacteria; p__Bacteroidetes; c__Flavobacteriia; o__Flavobacteriales; f__Flavobacteriaceae; |
|  | OTU_14727 | 15.15 | k__Bacteria; p__Bacteroidetes; c__Flavobacteriia; o__Flavobacteriales; f__Flavobacteriaceae; |
|  | OTU_49613 | 6.42 | k__Bacteria; p__Bacteroidetes; c__Bacteroidia; o__Bacteroidales; |
|  | OTU_46277 | 6.08 | k__Bacteria; p__Bacteroidetes; c__Flavobacteriia; o__Flavobacteriales; f__Flavobacteriaceae; |
|  | OTU_111946 | 2.90 | k__Bacteria; p__Bacteroidetes; c__Flavobacteriia; o__Flavobacteriales; f__Flavobacteriaceae; |
|  | OTU_2782 | 1.96 | k__Bacteria; p__Bacteroidetes; c__Flavobacteriia; o__Flavobacteriales; |
|  | OTU_36804 | 1.71 | k__Bacteria; p__Bacteroidetes; c__Flavobacteriia; o__Flavobacteriales |
|  | OTU_124947 | 1.66 | k__Bacteria; p__Bacteroidetes; c__Flavobacteriia; o__Flavobacteriales; f__Flavobacteriaceae; |
|  | OTU_102182 | 1.25 | k__Bacteria; p__Bacteroidetes; c__Flavobacteriia; o__Flavobacteriales; f__Flavobacteriaceae; |
|  | OTU_26687 | 1.22 | k__Bacteria; p__Bacteroidetes; c__Flavobacteriia; o__Flavobacteriales; f__Flavobacteriaceae; |
| *Actinia equina* | OTU_114561 | 24.31 | k__Bacteria; p__Bacteroidetes; c__Flavobacteriia; o__Flavobacteriales; f__Flavobacteriaceae; |
|  | OTU_14727 | 18.38 | k__Bacteria; p__Bacteroidetes; c__Flavobacteriia; o__Flavobacteriales; f__Flavobacteriaceae; |
|  | OTU_49613 | 11.37 | k__Bacteria; p__Bacteroidetes; c__Bacteroidia; o__Bacteroidales; |
|  | OTU_36804 | 9.40 | k__Bacteria; p__Bacteroidetes; c__Flavobacteriia; o__Flavobacteriales; |
|  | OTU_24017 | 3.10 | k__Bacteria; p__Bacteroidetes; c__Bacteroidia; o__Bacteroidales; f__Marinilabiaceae; |
|  | OTU_20001 | 2.36 | k__Bacteria; p__Bacteroidetes; c__Flavobacteriia; o__Flavobacteriales; f__Flavobacteriaceae; |
|  | OTU_72528 | 2.12 | k__Bacteria; p__Bacteroidetes; c__Flavobacteriia; o__Flavobacteriales; f__Flavobacteriaceae; |
|  | OTU_51932 | 1.99 | k__Bacteria; p__Bacteroidetes; c__Flavobacteriia; o__Flavobacteriales; f__Flavobacteriaceae; |
|  | OTU_175272 | 1.92 | k__Bacteria; p__Bacteroidetes; c__Flavobacteriia; o__Flavobacteriales; |
|  | OTU_122511 | 1.77 | k__Bacteria; p__Bacteroidetes; c__Flavobacteriia; o__Flavobacteriales; f__Cryomorphaceae; g__Owenweeksia; |
| *Holothuria forskali* | OTU_14727 | 18.48 | k__Bacteria; p__Bacteroidetes; c__Flavobacteriia; o__Flavobacteriales; f__Flavobacteriaceae; |
|  | OTU_114561 | 16.49 | k__Bacteria; p__Bacteroidetes; c__Flavobacteriia; o__Flavobacteriales; f__Flavobacteriaceae; |
|  | OTU_49613 | 9.98 | k__Bacteria; p__Bacteroidetes; c__Bacteroidia; o__Bacteroidales; |
|  | OTU_46277 | 6.67 | k__Bacteria; p__Bacteroidetes; c__Flavobacteriia; o__Flavobacteriales; f__Flavobacteriaceae; |
|  | OTU_2782 | 2.27 | k__Bacteria; p__Bacteroidetes; c__Flavobacteriia; o__Flavobacteriales; |
|  | OTU_36804 | 2.03 | k__Bacteria; p__Bacteroidetes; c__Flavobacteriia; o__Flavobacteriales; |
|  | OTU_111946 | 1.89 | k__Bacteria; p__Bacteroidetes; c__Flavobacteriia; o__Flavobacteriales; f__Flavobacteriaceae; |
|  | OTU_132862 | 1.83 | k__Bacteria; p__Bacteroidetes; c__[Rhodothermi]; o__[Rhodothermales]; f__[Balneolaceae]; g__KSA1 |
|  | OTU_51932 | 1.59 | k__Bacteria; p__Bacteroidetes; c__Flavobacteriia; o__Flavobacteriales; f__Flavobacteriaceae; |
|  | OTU_124947 | 1.23 | k__Bacteria; p__Bacteroidetes; c__Flavobacteriia; o__Flavobacteriales; f__Flavobacteriaceae; |
| *Holothuria tubulosa* | OTU_20843 | 14.81 | k__Bacteria; p__Bacteroidetes; c__Flavobacteriia; o__Flavobacteriales; f__Flavobacteriaceae; |
|  | OTU_74167 | 4.91 | k__Bacteria; p__Bacteroidetes; c__Bacteroidia; o__Bacteroidales; f |
|  | OTU_14727 | 3.37 | k__Bacteria; p__Bacteroidetes; c__Flavobacteriia; o__Flavobacteriales; f__Flavobacteriaceae; |
|  | OTU_114561 | 2.99 | k__Bacteria; p__Bacteroidetes; c__Flavobacteriia; o__Flavobacteriales; f__Flavobacteriaceae; |
|  | OTU_180399 | 1.71 | k__Bacteria; p__Bacteroidetes; c__Bacteroidia; o__Bacteroidales; |
|  | OTU_51932 | 1.48 | k__Bacteria; p__Bacteroidetes; c__Flavobacteriia; o__Flavobacteriales; f__Flavobacteriaceae; |
|  | OTU_8404 | 1.18 | k__Bacteria; p__Bacteroidetes; c__Cytophagia; o__Cytophagales; f__Flammeovirgaceae; g__Roseivirga; |
|  | OTU_134155 | 1.17 | k__Bacteria; p__Bacteroidetes; c__Flavobacteriia; o__Flavobacteriales; f__Flavobacteriaceae; g__Flavobacterium; |
|  | OTU_93736 | 1.04 | k__Bacteria; p__Bacteroidetes; c__Cytophagia; o__Cytophagales; f__Flammeovirgaceae; g__Reichenbachiella; |
|  | OTU_124311 | 1.01 | k__Bacteria; p__Bacteroidetes; c__Cytophagia; o__Cytophagales; f__Flammeovirgaceae; |
| *H. tubulosa* and *H. forskali* feces | OTU_72734 | 37.56 | k__Bacteria; p__Bacteroidetes; c__[Rhodothermi]; o__[Rhodothermales]; f__[Balneolaceae]; g__Balneola; |
|  | OTU_123527 | 21.66 | k__Bacteria; p__Actinobacteria; c__Actinobacteria; o__Actinomycetales; f__Microbacteriaceae; |
|  | OTU_20849 | 11.26 | k__Bacteria; p__Actinobacteria; c__Actinobacteria; o__Actinomycetales; f__Microbacteriaceae; g__Rathayibacter; s__caricis |
|  | OTU_132862 | 7.06 | k__Bacteria; p__Bacteroidetes; c__[Rhodothermi]; o__[Rhodothermales]; f__[Balneolaceae]; g__KSA1; |
|  | OTU_125434 | 4.21 | k__Bacteria; p__Actinobacteria; c__Actinobacteria; o__Actinomycetales; |
|  | OTU_152215 | 2.80 | k__Bacteria; p__Bacteroidetes; c__[Rhodothermi]; o__[Rhodothermales]; f__[Balneolaceae]; g__Balneola |
|  | OTU_105452 | 1.60 | k__Bacteria; p__Actinobacteria; c__Actinobacteria; o__Actinomycetales; f__Microbacteriaceae; |
|  | OTU_81589 | 1.31 | k__Bacteria; p__Actinobacteria; c__Actinobacteria; o__Actinomycetales; f__Microbacteriaceae; |
|  | OTU_43480 | 1.08 | k__Bacteria; p__Bacteroidetes; c__[Rhodothermi]; o__[Rhodothermales]; f__[Balneolaceae]; g__Balneola; |
|  | OTU_29763 | 1.05 | k__Bacteria; p__Bacteroidetes; c__[Rhodothermi]; o__[Rhodothermales]; f__[Balneolaceae]; g__KSA1; |
| Seawater from the aquaculture tank | OTU_31477 | 14.38 | k__Bacteria; p__Bacteroidetes; c__Flavobacteriia; o__Flavobacteriales; f__Flavobacteriaceae; |
|  | OTU_36812 | 13.10 | k__Bacteria; p__Bacteroidetes; c__Flavobacteriia; o__Flavobacteriales; f__Flavobacteriaceae; |
|  | OTU_14727 | 5.49 | k__Bacteria; p__Bacteroidetes; c__Flavobacteriia; o__Flavobacteriales; f__Flavobacteriaceae; |
|  | OTU_106047 | 2.99 | k__Bacteria; p__Bacteroidetes; c__Flavobacteriia; o__Flavobacteriales; f__Flavobacteriaceae; |
|  | OTU_119217 | 2.72 | k__Bacteria; p__Bacteroidetes; c__Flavobacteriia; o__Flavobacteriales; f__Flavobacteriaceae; |
|  | OTU_25130 | 2.36 | k__Bacteria; p__Bacteroidetes; c__Flavobacteriia; o__Flavobacteriales; f__Flavobacteriaceae; |
|  | OTU_2782 | 2.22 | k__Bacteria; p__Bacteroidetes; c__Flavobacteriia; o__Flavobacteriales; |
|  | OTU_69143 | 2.07 | k__Bacteria; p__Bacteroidetes; c__Flavobacteriia; o__Flavobacteriales; f__Flavobacteriaceae; |
|  | OTU_162823 | 1.91 | k__Bacteria; p__Bacteroidetes; c__Flavobacteriia; o__Flavobacteriales; f__Cryomorphaceae; |
|  | OTU_175272 | 1.69 | k__Bacteria; p__Bacteroidetes; c__Flavobacteriia; o__Flavobacteriales; |
