# Supplementary material for: Diversity and antimicrobial potential in sea anemone and holothurian microbiomes
Source: PLoS One. 2018 May 9;13(5):e0196178. doi: 10.1371/journal.pone.0196178 (PMC5942802; doi:10.1371/journal.pone.0196178)
Supplement: S4 Table — (DOCX) [file pone.0196178.s011.docx]

| Samples | Null  (Brokenstick) | Preemption | Lognormal | Zipf | Zipf-Mandelblot |
| --- | --- | --- | --- | --- | --- |
| *Anemonia sulcata* | 264344.70 | 243255.53 | 43937.08 | 19049.75 | 19051.75 |
| *Actinia* *equina* | 10483.52 | 5817.72 | 1597.23 | 1263.89 | 986.12 |
| *Holothuria* *forskali* | 2618097.00 | 2089410.84 | 190836.44 | 77027.96 | 77029.96 |
| *Holothuria* *tubulosa* | 1964675.00 | 1924186.00 | 312925.77 | 111107.11 | 111109.11 |
| Holothurian feces | 47856.40 | 27998.90 | 4507.14 | 2462.78 | 2207.72 |
| Seawater | 126554.00 | 85931.53 | 12160.22 | 9821.64 | 7588.77 |
